# Levels and Patterns of Genetic Diversity and Population Structure in Domestic Rabbits

**DOI:** 10.1371/journal.pone.0144687

**Published:** 2015-12-21

**Authors:** Joel M. Alves, Miguel Carneiro, Sandra Afonso, Susana Lopes, Hervé Garreau, Samuel Boucher, Daniel Allain, Guillaume Queney, Pedro J. Esteves, Gerard Bolet, Nuno Ferrand

**Affiliations:** 1 CIBIO, Centro de Investigação em Biodiversidade e Recursos Genéticos, Campus Agrário de Vairão, Universidade do Porto, Vairão, Portugal; 2 Department of Genetics, University of Cambridge, Cambridge, United Kingdom; 3 Departamento de Biologia, Faculdade de Ciências, Universidade do Porto, Porto, Portugal; 4 INRA, UMR1388 Génétique, Physiologie et Systèmes d’Elevage, Castanet-Tolosan, France; 5 Labovet Conseil (réseau Cristal), Les Herbiers, France; 6 ANTAGENE, Wildlife Genetics Laboratory, Limonest (Lyon), France; 7 CITS, Centro de Investigação em Tecnologias da Saúde, IPSN, CESPU, Gandra, Portugal; Embrapa, BRAZIL

## Abstract

Over thousands of years humans changed the genetic and phenotypic composition of several organisms and in the process transformed wild species into domesticated forms. From this close association, domestic animals emerged as important models in biomedical and fundamental research, in addition to their intrinsic economical and cultural value. The domestic rabbit is no exception but few studies have investigated the impact of domestication on its genetic variability. In order to study patterns of genetic structure in domestic rabbits and to quantify the genetic diversity lost with the domestication process, we genotyped 45 microsatellites for 471 individuals belonging to 16 breeds and 13 wild localities. We found that both the initial domestication and the subsequent process of breed formation, when averaged across breeds, culminated in losses of ~20% of genetic diversity present in the ancestral wild population and domestic rabbits as a whole, respectively. Despite the short time elapsed since breed diversification we uncovered a well-defined structure in domestic rabbits where the F_ST_ between breeds was 22%. However, we failed to detect deeper levels of structure, probably consequence of a recent and single geographic origin of domestication together with a non-bifurcating process of breed formation, which were often derived from crosses between two or more breeds. Finally, we found evidence for intrabreed stratification that is associated with demographic and selective causes such as formation of strains, colour morphs within the same breed, or country/breeder of origin. These additional layers of population structure within breeds should be taken into account in future mapping studies.

## Introduction

The European rabbit (*Oryctolagus cuniculus*) is the sole progenitor of domestic rabbits and both the wild and domestic forms occur worldwide.

Its geographic origin can be traced back to Iberian Peninsula [1,2], where two subspecies coexist: *O*. *c*. *cuniculus*, which is distributed in the north-eastern portion of the Iberian Peninsula, and *O*. *c*. *algirus*, on the south-western part [3]. After the last glacial maximum, ~18,000 years ago, the European rabbit crossed the Pyrenees and expanded its natural range to the South of France [4,5]. The following worldwide expansion was human-mediated with its most important period during the Middle Ages [1,6,7].

Humans have hunted rabbits for thousands of years, but their domestication did not happen until recent times. The historical record reveals contradictory information regarding the geographic origin of domestic rabbits. Some records indicate that the initial steps took place in the Iberian Peninsula in the first century BC, where Romans raised rabbits for meat and fur in large fenced colonies. However, this practice is most likely to have occurred without selective breeding [6,8]. Alternatively, there is abundant historical information indicating that the true domestication process, including taming and selective breeding, occurred in French Monasteries within the last 1,500 years. Presumably this was inadvertently triggered by the Pope Gregory I, when in 600 AD decreed that *Laurices*, a delicacy consisting of unborn or newly born rabbits, were not considered meat and thus suitable for eating during Lent and other fasts [8–10]. French monks that lived in seclusion and needed easily obtainable meat supplies, found in this permission a motivation to initiate the selective breeding of rabbits.

Genetic data supports a domestication origin in France and shows that domestic rabbits display a subset of the genetic variability found in the *O*. *c*. *cuniculus* French wild populations [4,7,11–15]. The establishment of most rabbit breeds was more recent and it began at the end of the 18th century in Western Europe [7,10,16]. However, breeds of various sizes and colours were reported in the 16th century suggesting that the diversification process may have started earlier [16]. Currently, there are more than 200 rabbit breeds and strains, which have been selected for a wide range of purposes (*e*.*g*. meat, wool, fur, therapeutic proteins, companion animals) [10,17]. These breeds exhibit an immense phenotypic diversity for a variety of traits, which has surprisingly accumulated in the short period of time since the initial domestication. Moreover, domestic rabbits share a large number of genetic diseases with humans (*e*.*g*. hypertension, epilepsy, osteoporosis), making them valuable models in biomedical research [17,18].

The genetic studies so far have focused on a small number of breeds [13–15] or genetic markers [4,7,13,19], therefore patterns of population structure within domestic rabbits remain poorly characterized.

Here, by taking advantage of the availability of wild rabbit populations, we investigated the domestication history of rabbits and quantified the amount of genetic diversity lost with the initial domestication process as well as with the more recent process of breed formation. In addition, we assessed genetic relatedness and substructure among rabbit breeds.

## Material and Methods

### Ethics Statement

No ethical approval was deemed necessary for this study. The European rabbit is a game animal in all locations sampled and all tissue samples for the wild animals were donated by local associations of hunters, who held appropriate permits for hunting wild rabbit in season. The fieldwork did not involve any endangered or protected species. No experimental studies in live animals were performed in this study. The sampling of blood from domestic animals for DNA extraction was approved and performed at INRA (Institut National de la Recherche Agronomique, France) by specialised veterinaries, and in accordance with the national regulations for the use of animals in agriculture. No animals were purposely killed for this study.

### Sample Selection

To obtain a representative sample of the genetic diversity captured with rabbit domestication we followed three criteria. First, given that historical records indicate that most modern breeds were derived from crosses among ancient breeds [10], we sampled breeds that are known to have an old origin because these are likely to represent genetic diversity reservoirs. Second, to further help capturing a broad range of the genetic diversity present in the domesticated population we sampled breeds originating from distinct geographic regions. Finally, we focused on a subset of breeds representing the most divergent phenotypic characteristics, assuming that high phenotypic divergence may, in principle, reflect higher genetic divergence. The following 16 different breeds were used: Belgian Hare, Champagne Silver, Chinchilla (Standard), English Spot, English Silver, Fauve de Bourgogne, French Angora, French Lop, Flemish Giant, Himalayan, Hungarian Giant, New Zealand, Netherland Dwarf, Rex, Thuringer, and Vienna White (S1 Table). The breeds New Zealand and Rex are both represented by different strains. We sampled New Zealand individuals belonging to the strains INRA 1077 (a line selected for litter size) and INRA 9077 (a control line maintained without selection). The Rex breed comprises three colour morphs (they are referred to in this study as Castor, Chinchilla and White Strain). The White Strain was obtained by controlled crossings between the New Zealand and the Chinchilla Rex Strain.

The choice of wild rabbit samples was based on molecular and historical information regarding the most plausible route that led to the domestication of the European rabbit (see Introduction). We surveyed individuals belonging to the subspecies *O*. *c*. *cuniculus* from four localities in the northeastern part of the Iberian Peninsula and nine localities in France (S2 Table and S1 Fig). In total, and considering both wild and domestic animals, we sampled 471 individuals and divided them into three main groups: wild rabbits from Iberia (n = 39), wild rabbits from France (n = 92) and domestic rabbits (n = 340). In several analyses presented below the domestic group was further subdivided into breeds or strains. Total genomic DNA was extracted from blood using high salt or phenol-chloroform standard protocols [20].

### Selection of Molecular Markers

To determine levels and patterns of genetic variation we used a total of 45 microsatellites distributed throughout the rabbit genome (S3 Table). We chose 38 microsatellites located on the autosomes and 7 microsatellites located on the X-chromosome. The majority of the microsatellites used in this study (n = 33) were obtained from previous studies [21,22], but 12 additional microsatellites were developed in order to increase the number of loci and provide a more even representation across the genome. These additional microsatellites were identified using the rabbit reference genome sequence (OryCun2; http://www.ensembl.org) and their genomic location is given on S4 Table. With the exception of STR06 that was characterized by a tetranucleotide motif, all other microsatellites were composed of dinucleotide motifs.

### Microsatellite Genotyping

Primer pairs were designed using the web-based version of Primer3plus (http://www.bioinformatics.nl/cgi-bin/primer3plus/primer3plus.cgi) (S5 Table) and screened for primer-dimer interactions using the AUTODIMER software [23]. Multiplex panels were designed taking into account these interactions and expected lengths of the different amplicons. Nine independent multiplex reactions using fluorescent-labeled primers were used. PCRs were prepared in a total reaction volume of 5 μL, containing Qiagen PCR Master Mix (Qiagen, Valencia, CA), primer mix (*i*.*e*. solution containing all the primers and dyes in variable concentrations), water and genomic DNA. PCR products were separated by capillary electrophoresis using an ABI Prism 3130xl, automated sequencer and Genescan-500 LIZ as size standard (PE Applied Biosystems, Foster City, CA). Genotypes were scored with the software GENEMAPPER 4.0 (PE Applied Biosystems, Foster City, CA), followed by visual inspection and manual corrections whenever required. Microsatellite genotypes per marker and for each individual sample are available in S1 File. Multiplex information and PCR conditions are described in detail in S6 and S7 Tables.

For each microsatellite, programs MICRO-CHECKER version 2.2.3 [24] and ARLEQUIN version 3.5.1.2 [25] were used to detect the potential presence of null alleles and deviations from Hardy-Weinberg equilibrium. Both tests were performed in the wild populations composed of more than 8 individuals because these are more likely to represent random mating populations. With the exception of STR22 and STR25, we found no systematic evidence for the presence of null alleles and deviations from Hardy-Weinberg equilibrium for the remaining loci. Because the results remained qualitatively unaltered including or excluding these microsatellites (data not shown), the analyses presented below include the full dataset of 45 microsatellites.

### Data analysis

#### Summary statistics of genetic diversity and differentiation

Sample size for each locus (n), number of alleles (Na), expected heterozygosity (*He*) and Fixation Index (*FIS*) were calculated using the program GENALEX 6.4.1 [26]. Allelic richness (*Ar*) and Private Allelic Richness (*PAr*) were estimated using the program HP-RARE 1.0 [27], using a rarefaction strategy to accommodate differences in sample size between groups. Genetic differentiation among domestic breeds was estimated using both global and pairwise F_ST_ [28] and calculated with ARLEQUIN. The same program was also used to investigate the partition of genetic variability by performing a hierarchical analysis of molecular variance–AMOVA [29]. This analysis was conducted independently for domestic breeds and wild populations, while considering genetic variation as a three-level structure (*i*.*e*. among breeds/localities, among individuals within breeds/localities, and within breeds/localities). Statistical significance of results was evaluated with 10,000 permutations.

#### Phylogenetic analysis

Phylogenetic trees of individuals were constructed using Chord-distances [30] and the allele- sharing distance [31] by means of the computer program POPULATIONS version 1.2.31 [32]. Both distances have been widely applied to the study of domesticated species [33,34]. Due to the large number of individuals sampled, we used the neighbour-joining algorithm due to its efficient computational speed [35,36]. The resulting tree composed by all individuals was re-rooted with the cluster composed by wild rabbits from Iberian Peninsula for visualisation. To evaluate genetic relationships between breeds, instead of obtaining genetic distances among individuals, distances were generated among breeds and the wild French population (*i*.*e*. localities were collapsed into a single group). The software MICROSATELLITE ANALYSER (MSA) version 4.05 [37] was used to replicate 1000 genetic distance matrices. Given the lower number of entries compared to the analysis focused on individual animals, we used the Fitch-Margoliash least-squares algorithm, which has the advantage of being more accurate at the cost of slower computational speed [36,38]. Phylogenetic trees of populations were constructed for each of the 1000 replicated datasets using the program FITCH with global rearrangements and the input sequence order randomized. The program CONSENSE was used to create a majority-rule consensus tree using wild Iberian rabbits as outgroup. Both programs are implemented in the computer package PHYLIP version 3.69 [36]. Final trees were drawn and edited using FigTree, version 1.3.1, (http://tree.bio.ed.ac.uk/software/figtree/).

#### Population structure

Genetic structure within the domesticated population was investigated using two distinct approaches. First, we applied the Bayesian clustering procedure implemented in STRUCTURE, version 2.3.3 [39]. Five independent runs with values of *K* (*i*.*e*. potential number of genetic clusters) ranging from 1 to 30 were performed for a dataset composed of all domestic rabbits. We chose a broad interval of *K* to i) infer potential ancestral relations among breeds by exploring clustering patterns resulting from lower values of *K*, and ii) investigate the existence of potential substructure within breeds by focusing on higher values of *K*. Each run was carried out with 100,000 iterations following a “burn-in” period of 50,000 iterations. The analysis was performed using the admixture model and assuming that allele frequencies are correlated among populations. After the first analysis including all domestic animals, a second analysis was conducted independently for each breed in order to evaluate the existence of additional substructure within breeds. The most probable *K* was inferred using the method proposed by Evanno *et al* [40]. This estimation was performed with the online version of STRUCTURE HARVESTER [41] The graphical display of the STRUCTURE results was generated using DISTRUCT software [42]. CLUMPP software [43] was used to deal with label switching within each run of a different *K*, using the LargeKGreedy algorithm and 10000 random input sequences. Consistency among runs assuming the same value of *K* was evaluated using a similarity coefficient measure (C) as described by Rosenberg [44] and calculated using the package SIMCO for the R statistical environment (R Development Core Team, 2012).

Second, we conducted a Discriminant Analysis of Principal Components–DAPC [45]. In contrast to other common multivariate approaches (*e*.*g*. Principal Component Analysis–PCA, or Factorial Correspondence Analysis–FCA), DAPC maximizes the separation between groups while minimizing variation within a group, providing a better discrimination of pre-defined genetic groups. Moreover, it allows obtaining a graphical representation of the relationships between the inferred clusters. This method has also the advantage of not relying on a particular population genetics model, such as Hardy-Weinberg equilibrium and linkage disequilibrium assumptions [45], a key assumption of the algorithm implemented in STRUCTURE. In the case of domestic populations this is particularly relevant, since they often show violations to the Hardy-Weinberg equilibrium due to non-random mating. We carried out the DAPC analysis using the different breeds as pre-defined groups, and using the software ADEGENET package [46] implemented in R [47]. For the preliminary data transformation step we retained a number of principal components sufficient to explain ~90% of the cumulative variance. In the discrimination analysis five discriminant functions were retained.

Finally, we conducted a population assignment test among the 16 domestic breeds with GENALEX according to the approach of Paetkau *et al* [48].

## Results

### Genetic relationships between wild and domestic animals

**Fig 1:**
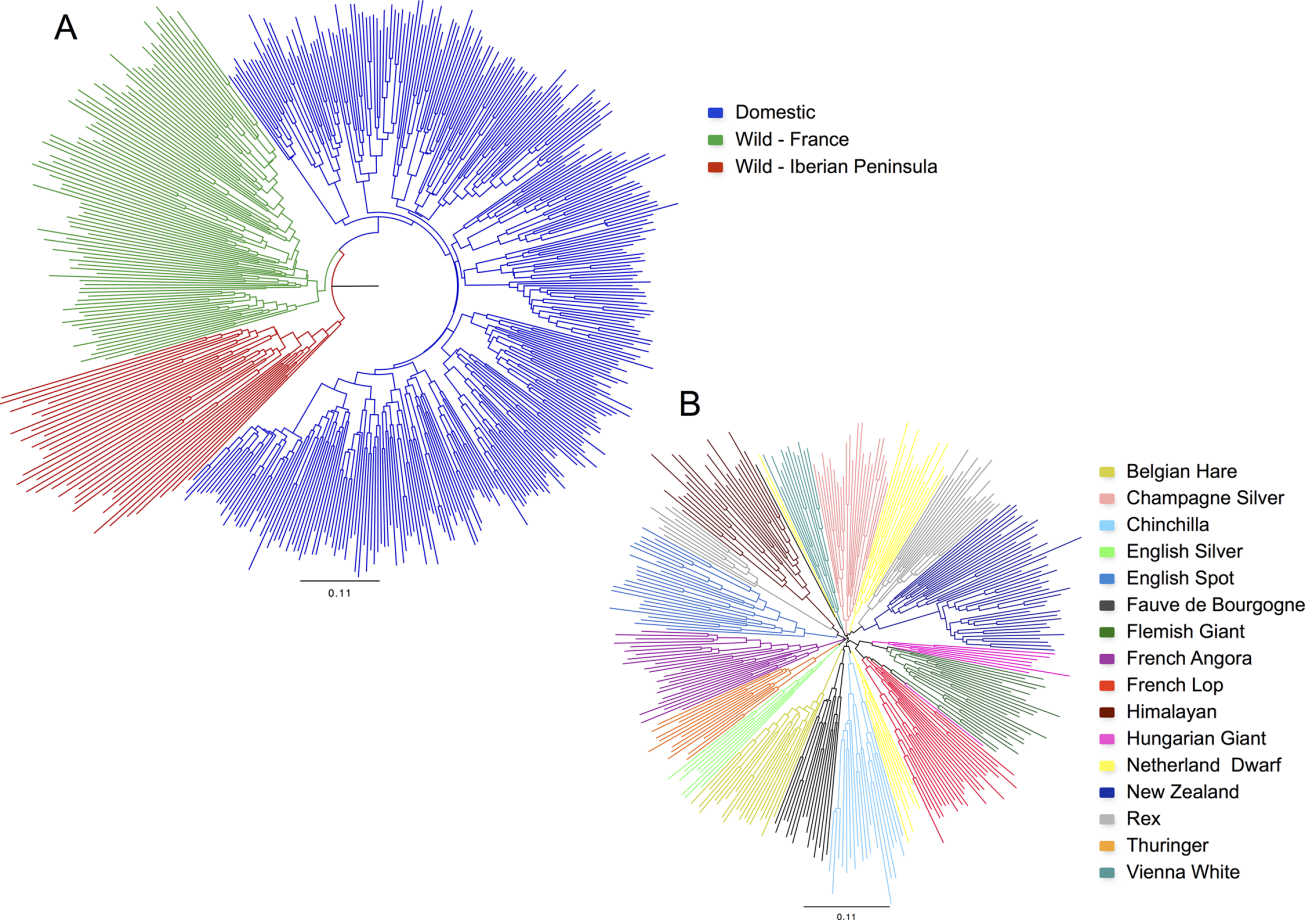
Phylogenies of wild and domestic rabbits based on chord genetic distance. Trees were constructed using all 45 microsatellites. Branches are coloured according to individual’s origin. (A) Neighbour-joining tree for 471 individuals rooted with wild rabbits from the Iberian Peninsula. (B) Unrooted Neighbour-joining tree for 340 domestic individuals from 16 different breeds.

To investigate the relationships between wild and domestic animals we conducted a phylogenetic analysis based upon all 471 individuals. The resulting topology distinguishes the three major population groups considered in this study (Fig 1A). Wild rabbits from France grouped within the genetic pool present in Iberian Peninsula, in accordance with previous studies that suggest wild rabbits from Iberia as the source of French wild rabbits [4,5]. All domestic individuals in turn clustered together within the genetic pool of French wild rabbits. This proximity is also supported by F_ST_ values averaged across all markers that indicated less differentiation between these two populations (F_ST_ = 12%) than between domestic rabbits and wild rabbits from the Iberian Peninsula (F_ST_ = 16%). An alternative phylogeny based on a different genetic distance (allele-sharing distance) produced qualitatively similar results with the exception of the population of Alicante that did not group together with the remaining Iberian populations (S2A Fig).

### Levels and patterns of microsatellite genetic diversity and differentiation

Summary statistics of genetic diversity are presented in Table 1 and S8 Table. An overview of the global diversity patterns across the three groups showed a directional loss of genetic diversity from wild rabbits from the Iberian Peninsula to France, and then to domestic rabbits, which was consistent across the majority of the loci analysed. Regarding the expected heterozygosity (*He*), wild rabbits from the Iberian Peninsula exhibited the highest levels of genetic diversity averaged across loci (*He* = 0.825), followed by French wild rabbits (*He* = 0.723), and finally domestic rabbits (*He* = 0.581). Allelic and private allelic richness followed similar trends. These reductions in genetic diversity were statistically significant for all summary statistics (P < 0.001, two-tailed Wilcoxon signed rank test). This pattern is consistent with the occurrence of population bottlenecks in the colonization of France and in the initial domestication process. We did not find significant differences between levels of genetic diversity for loci residing on the autosomes vs. X-chromosome (P > 0.10, Mann-Whitney).

Estimates of genetic diversity observed within the different breeds were similar and generally low, suggesting additional bottlenecks in the process of breed formation. The average expected heterozygosity within breeds was 0.457 and allelic richness (corrected for a minimum sample size of 8 genes) averaged 2.39. Values of private allelic richness were also low and averaged 0.06. An analysis of molecular variance (AMOVA) was conducted for three distinct groups: 1) four sampling localities from Iberian Peninsula; 2) nine sampling localities from France; and 3) 16 domestic breeds (Table 2). Domestic rabbits had the highest percentage of variation among populations (~22%), followed by wild rabbits from France (~14%) and finally wild rabbits from the Iberian Peninsula (~7%). These values show that even though most domestic breeds were formed in the last 200 years, they tend to be more differentiated amongst themselves than localities within Iberian Peninsula and France separated by large geographic distances. Most of the variation in all comparisons, however, was found within individuals, with French rabbits being the most variable, followed by Iberian rabbits and finally domestic rabbits. The global F_ST_ among breeds was 22%, with values ranging from 9% to 41% for the Flemish Giant/Hungarian Giant and Belgian Hare/English Spot comparisons, respectively. We found statistical support for genetic differentiation in all pairwise F_ST_ comparisons among breeds (P < 0.001; S9 Table).

**Table 1:** Measures of genetic diversity for groups and breeds.

		Strains	*n ^a^*	*Na ^b^*	*Ar ^c^*	*Par ^d^*	*He ^e^*
**Groups**	Wild (Iberian Peninsula)		39	11.044	10.68	4.11	0.825
	Wild (France)		92	8.444	7.27	0.67	0.723
	Domestic		340	6.356	4.80	0.15	0.581
**Breeds**	Belgian Hare		21	2.822	2.14	0.03	0.369
	Champagne Silver		25	3.378	2.45	0.06	0.465
	Chinchilla		20	3.178	2.51	0.10	0.490
	English Silver		8	2.667	2.39	0.05	0.479
	English Spot		25	3.000	2.24	0.04	0.426
	Fauve de Bourgogne		16	3.178	2.42	0.07	0.462
	Flemish Giant		25	3.222	2.35	0.05	0.442
	French Angora		25	3.200	2.39	0.07	0.457
	French Lop		25	3.578	2.60	0.10	0.498
	Himalayan		23	3.244	2.37	0.05	0.434
	Hungarian Giant		8	2.933	2.52	0.04	0.463
	Netherland Dwarf		25	3.644	2.60	0.08	0.489
	New Zealand		42	3.111	2.28	0.07	0.435
		INRA 1077	24	2.089	1.78	0.03	0.305
		INRA 9077	18	2.800	2.39	0.07	0.469
	Rex		25	3.333	2.33	0.05	0.505
		Castor	9	2.000	1.90	0.00	0.357
		Chinchilla	9	2.311	2.04	0.02	0.372
		White	8	2.489	2.29	0.02	0.439
	Thuringer		13	2.778	2.28	0.05	0.436
	Vienna White		14	2.911	2.39	0.02	0.460
***Mean*** *				3.136	2.39	0.06	0.457
***SE*** *				0.069	0.032	0.006	0.008

^a^ Number of individuals

^b^ Number of observed Alleles

^c^ Allelic Richness

^d^ Private Allelic Richness

^e^ Expected Heterozygosity

* Values for breeds only. The strains were not considered in the calculation.

**Table 2:** Analysis of Molecular Variance for domestic breeds and wild localities.

	Source of variation	d.f.	Sum of squares	Variance components	% of variation
**Domestic**	Among breeds	15	1651.728	2.39515 Va	22.2
	Among individuals within breeds	324	3010.446	0.94281 Vb	8.78
	Within individuals	340	2518	7.40588 Vc	68.93
	*Total*	679	7180.174	10.74384	
**Wild French**	Among localities	8	358.409	1.79126 Va	14.14
	Among individuals within localities	83	892.053	-0.13162 Vb	-1.04
	Within individuals	92	1013	11.01087 Vc	86.9
	*Total*	183	2263.462	12.67051	
**Wild Iberian**	Among localities	3	120.019	1.14629 Va	6.78
	Among individuals within localities	35	627.994	2.17006 Vb	12.83
	Within individuals	39	530.5	13.60256 Vc	80.4
	*Total*	77	1278.513	16.91891	

d.f., degrees of freedom.

All values of variance components have significant differences from zero at P < 0.0001.

### Loss of genetic diversity with the domestication and breed formation processes

To more formally estimate the amount of genetic diversity lost by the i) colonization of France, ii) initial domestication process, and iii) the process of breed formation, we conducted a resampling procedure following Gray *et al* [49]. This procedure has the advantage of reducing biases caused by unequal sample sizes between groups, sampling of closely related individuals, or inbreeding. We summarized the loss of genetic diversity using *He*. The amount of diversity lost with the colonization of France was estimated by sampling one chromosome from each of the 9 wild French populations and 4 wild Iberian populations localities and calculated as 1 –(*He* in wild French) / (*He* in wild Iberian). The loss of diversity caused by the initial domestication process was estimated by sampling one chromosome from each of the 16 rabbit breeds and 9 wild French populations localities and calculated as 1 –(*He* in domestic) / (*He* in wild French). Finally, genetic diversity lost in the process of breed formation was estimated by sampling one chromosome from each individual in each breed and one chromosome for each individual across all domestic animals and calculated as 1 –(*He* in breed) /(*He* in domestic). We resampled 1,000 times per comparison. It should be noted that the levels of genetic diversity found in current wild populations from Iberian Peninsula and France were used as proxies for the amount of genetic diversity historically available in the wild populations involved in the colonization of France and initial domestication process, respectively.

**Fig 2:**
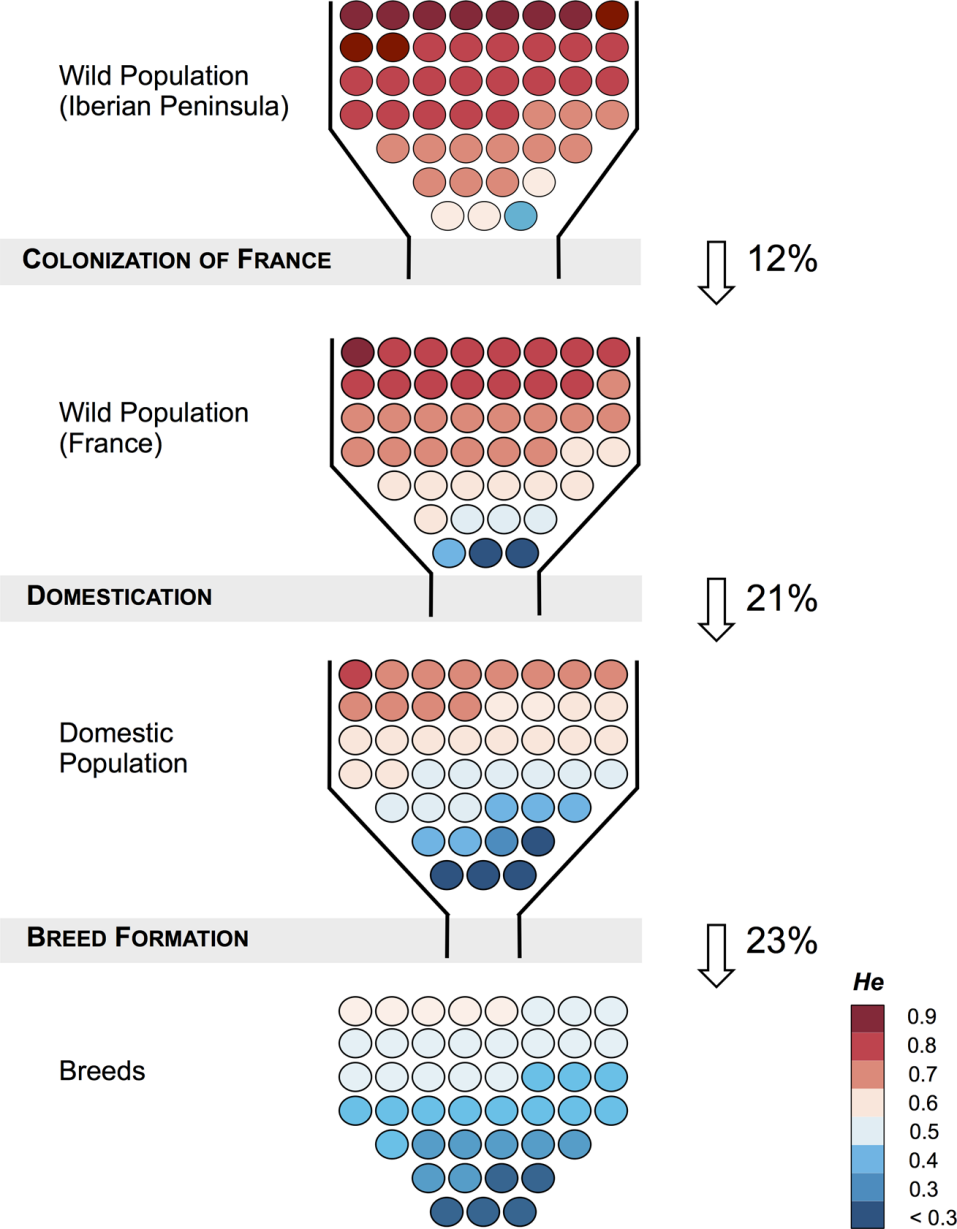
Reduction of genetic diversity along the rabbit domestication route. Each of the 45 circles represents a single microsatellite coloured according to the proportion of the expected heterozygosity per microsatellite present on its respective population. Note that the two wild samples (Iberian Peninsula and France) are proxies for the ancient wild samples that were involved in colonization of France and domestication. Shaded areas indicate the different bottleneck events that occurred at the colonization of France, initial domestication event, and breed formation. Values aside arrows show the amount of genetic diversity lost in each event estimated using a resampling methodology (See Methods).

Consistent with serial population contractions, we inferred a 12% (95% CI 4–20) decrease in genetic diversity with the colonization of France from the native range in Iberia, a decrease of 21% (95% CI 14–27) in the initial domestication process, and a decrease of 23% (95% CI 19–27) in the process of breed formation when averaged across breeds. (Fig 2, S3 Fig and S10 Table). For individual breeds the loss of genetic diversity ranged between 13% (95% CI 8–18) in Rex to 37% in Belgian Hare (95% CI 32–42).

### Genetic relationships among breeds and patterns of population structure within domestic rabbits

Using chord and allele-sharing distances between individuals, respectively, we constructed two different trees of individuals using only the domestic dataset (Fig 1B and S2B Fig). The trees inferred showed similar results and revealed a well-defined population structure within domestic rabbits. In most cases, individuals of a given breed grouped together, demonstrating that domestic rabbits are genetically more similar to individuals from their own breed rather than to individuals from other breeds.

To analyse in greater detail the relationships among breeds and to obtain support values, we constructed two additional consensus trees using both chord and allele-sharing distances among breeds instead of individuals (S4 Fig). Following the majority-rule criterion, we only considered relationships to be statistically supported if more than half of the replicates produced a similar topology. The resulting trees were consistent in showing with high statistical support that all breeds clustered together and that all domestic breeds are derived from wild French rabbits. Moreover, some relationships between breeds were in agreement with historical information regarding their origin. For example, the breeds Flemish Giant and Hungarian Giant clustered together with strong support, in accordance to historical records that indicate Flemish Giant as the source breed of the Hungary native breed [10]. Moreover, the two New Zealand strains were closely grouped with a high level of confidence as well as two of the three Rex strains (Chinchilla and White). Despite the lack of historical evidence, the strain Castor Rex and Himalayan were found to cluster with high statistical support, which can be indicative of a recently shared ancestry. It should be noted, however, that the inferred tree showed a generalized lack of statistical support for most branches. This limited power to infer genetic relationships among breeds may be a direct consequence of intrinsic aspects associated with rabbit domestication or a limitation of the methods and number of genetic markers used in this study (see Discussion).

**Fig 3:**
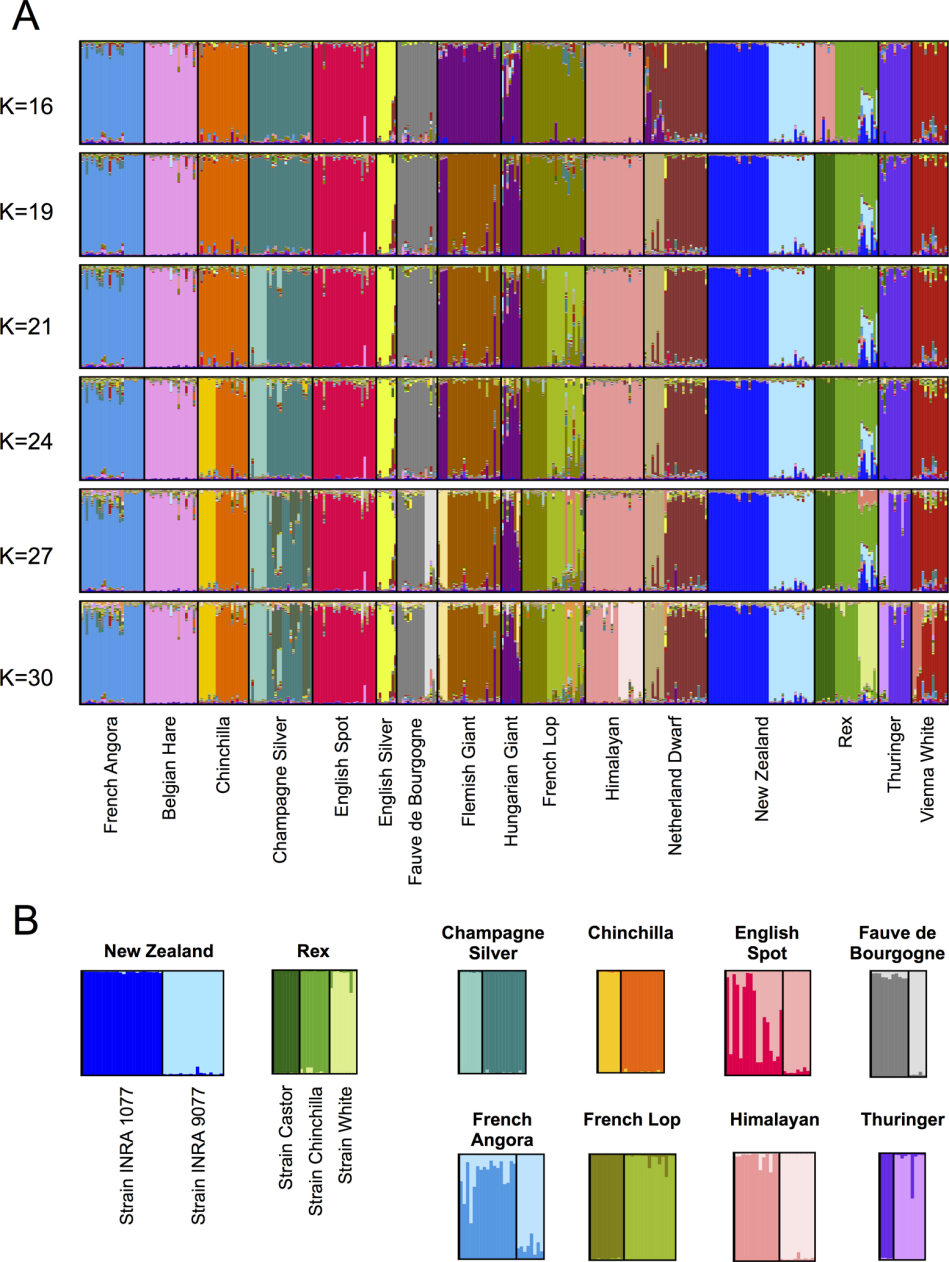
Individual assignment of domestic rabbits inferred with Bayesian cluster analysis (STRUCTURE). (A) Representative runs of 340 domestic individuals from 16 domestic breeds for different values of *K*. (B) Representative runs for 10 different breeds at *K* = 2 and *K* = 3 (exclusively for Rex).

Next, we investigated patterns of population structure within domestic rabbits by means of the clustering software STRUCTURE (Fig 3A). With this method, 340 samples from 16 domestic breeds were analysed with no prior information regarding their origin. The number of pre-defined clusters varied from 2 to 30 (plots of all runs are shown in S5 Fig). The choice of a broad *K* interval allowed us to investigate not only ancestral relationships between breeds (inferred by the clusters formed using low values of *K*) but also to analyse eventual substructure within breeds (using values of *K* higher than the total number of breeds).

The initial values of *K* exhibited very low consistency among runs, which may be indicative of a lack of deep structure within domestic rabbits. This observation was also supported by the low values of the similarity coefficient among the different runs, which we had to restrict to values of *K* below 9 due to computational limitations (S11 Table). With increasing values of *K*, replicate runs began to gain consistency (assessed visually), and when assuming a number of populations matching the total number of breeds sampled (*K* = 16), the structure plot shows a clear separation between most breeds (Fig 3A). Exceptions were only found for Flemish Giant and Hungarian Giant that share a cluster (likely a consequence of their recently shared history) and for the Castor Rex strain and Himalayan breed. The best likelihood, however, was found for *K* = 19, which is in conformity with the total number of breeds plus strains used in this study (*i*.*e*. 16 breeds, two of which composed by two or three strains) (S6 Fig). A population assignment test [48] also correctly assigned 99% of the individual rabbits (337 out of 340) to the respective breed of origin, which is likely a consequence of the well-defined breed structure.

In addition to the strong differentiation among breeds, we detected additional levels of substructure that caused the allocation of individuals of a same breed into separate clusters (Fig 3A). This additional substructure began to consistently emerge in higher values of *K* (*K* > 19) but in some cases preceded the separation of all breeds (*e*.*g* New Zealand and Rex strains; S5 Fig). These results were confirmed when the STRUCTURE analysis was carried out independently for each single breed (Fig 3B).

One of the STRUCTURE assumptions is that within populations the loci are at Hardy-Weinberg equilibrium (HW). Therefore, in addition to searching for microsatellites that showed significant deviations across all samples in wild populations (see Material and Methods) we used chi-square statistic to test deviations to HW for each marker in each breed (S12 Table). After applying a Bonferroni correction to account for multiple tests, we found a relatively low number of markers significantly deviated from HW equilibrium. Across all breeds we found on average 6.06% markers showing this departure, ranging from 0% for four breeds (*i*.*e*. English Spot, Hungarian Giant, Thuringer and Vienna White) and 19.05% for the New Zealand rabbits. As expected, the breeds more deviated from Hardy-Weinberg equilibrium markers are consistently those with high levels of intrabreed stratification. Even though the population structure patterns found are independently confirmed by a non-model approach (see DAPC below), one should take these results with caution, in particular due to the risk of overestimation of *K* [39].

The DAPC also provides membership probabilities of each individual belonging to a given cluster [45]. Similarly to STRUCTURE, the group memberships inferred with this non-model based approach revealed identifiable well-defined population structure in the domestic population with ~90% of the individuals being correctly assigned to the breed of origin. The visual assessment of between-population differentiation in the scatter plots was also consistent with a lack of deeper levels of structure above the breed level and the great majority of the inferred clusters were amalgamated at the centre of the plot area (S7 Fig). A slightly higher level of differentiation from the central group of breeds was detected for English Spot, New Zealand, Belgian Hare, and Angora.

## Discussion

### Similar losses of genetic diversity occurring at early domestication and breed formation

During most of its existence, the European rabbit was restricted to the Iberian Peninsula. Today, wild rabbits are distributed across Europe and new populations emerged worldwide as a result of human-mediated introductions [50,51]. The wild populations from France are an exception, and are likely the result of a natural expansion across the Pyrenees after the last glacial maximum [4,5]. This geographic expansion ultimately ended in one of the most recent domestication processes and the only one that occurred exclusively in Western Europe. Our data suggest that domestic rabbits form a homogenous group and are a subset of the genetic diversity found in French wild populations, which is in agreement with historical records [8–10] and previous genetic studies indicating a single origin of domestication in this region [7,14,15].

One of the main goals of our study was to investigate how the domestication process affected levels of genetic diversity in domestic rabbits (Fig 2). We found evidence for two major reductions of genetic diversity in their recent history, the first resulting from the derivation from wild populations and the second associated with breed formation. Using a resampling methodology, and the levels of genetic diversity present in extant wild populations as a surrogate estimate for the ancient levels of diversity present in the French wild populations that presumably gave origin to domestic rabbits, we estimate that the initial domestication accounted for losses of 21% of the pre-existing levels of genetic diversity in the wild. This estimate is similar to the one obtained by Queney *et al* [7] with six microsatellites (~18%), but differs from the value obtained by Carneiro *et al* [15], which estimated losses of 40% using sequence data. The discrepancy between these estimates may be caused by several non-mutually exclusive factors: 1) the higher mutation rate of microsatellites in comparison with nucleotide substitutions enabled a faster reconstitution of genetic diversity levels in domestic rabbits; 2) the larger number of individuals/ breeds used in this study may have maximized the amount of genetic diversity captured in domestic rabbits; and 3) different properties of the summary statistics used to estimate genetic diversity in microsatellites vs. sequencing data. Such a discrepancy between different molecular markers relative to the amount of genetic diversity lost with domestication has been observed before in other domestic species (*e*.*g* maize, wheat, sunflower) for which the microsatellite estimates of diversity loss are lower values than those derived from sequence data [52–54].

The similar reduction of genetic diversity with the domestication and breed formation is surprising given that the later is normally seen as a more direct and extreme process, where founder effects, population contraction and artificial selection should be stronger. Several non-mutually exclusive explanations may account for this observation. First and perhaps most importantly, rabbits seem to have a single origin of domestication. In fact, while many domestic animals emerged from multiple domestication events or originated from different wild species or subspecies (reviewed in [55,56]), domestic rabbits were derived from a single subspecies (*i*.*e*. *O*. *c*. *cuniculus*) and exclusively from the genetic pool available in France. Concurrently, the fact that rabbit domestication occurred in an historical time where many other domestic species already existed may have contributed to a more focused and incisive process that might in many ways have resembled the process of breed formation. Historical records suggest that the early rabbit domestication occurred in closed environments such as monasteries [8–10]. This implies relatively small population sizes, which may also have contributed to the severity of the rabbit domestication bottleneck. Finally, the occurrence of backcrosses with the wild ancestors has often been described for other mammalian species (*e*.*g*. dogs and pigs), a practice that can increase genetic diversity levels in the domesticated population [57]. Our results suggest that this practice may not have been as frequent in rabbits.

### Well-defined breed structure but absence of ancient structure in domestic rabbits

Domestic rabbits exhibit a clear and detectable genetic substructure. This is clearly demonstrated by several aspects of our data. For example, we inferred high and significant F_ST_ values among breeds (F_ST_ = 22%). This well-defined breed structure is also evident in the phylogenetic tree in which individuals of the same breed consistently grouped together (Fig 1B). The level of differentiation between rabbit breeds appears to be higher than that in many domestic mammals. For example, previous studies using microsatellites described F_ST_ values of ~6–13% between sheep breeds [58,59], 7–11% in cattle [60,61], ~8–12% in horses [62,63] and ~7% in goats [64]. The average value in domestic rabbits is more similar to values reported for pigs and dogs (~27%) [34,65]. Interestingly, the low number of private alleles detected among breeds suggests that this differentiation is mainly caused by changes in allele frequencies. Therefore, even though domestic rabbits share a common and very recent ancestry, the continuous and differential artificial selection, together with modern breeding practices that maintain breeds as closed genetic pools, were able to shape genetic diversity into strongly differentiated genetic compartments.

The well-defined breed structure contrasts with the phylogeny of breeds that showed a general lack of statistically supported subdivision in deeper branches (S4 Fig). This difficulty to resolve deeper levels of structure, other than structure associated with breeds, could be an intrinsic limitation of the used methods or may simply reflect the process of breed formation. For example, methods based on genetic distance matrices may lose information by collapsing individual genetic distances into a single piece of genetic information. However, an identical picture arises when we performed a clustering analysis using STRUCTURE and DAPC. These analyses showed that rabbit breeds are genetically distinct and can be separated on the basis of their genotype alone, but similarly, failed to detect deeper relationships among breeds in most cases. This is well illustrated by the lack of consistency among runs using lower values of *K* in the STRUCTURE analysis and the lack of identifiable structure in the scatter plot of the DAPC. Even though we mainly used European and North American rabbit breeds in the present study, both historical records [10] and genetic studies in other non-European breeds [66] have shown that domestic breeds worldwide were derived very recently from European breeds. Perhaps more likely, and as suggested by Parker *et al* [34] based on similar results in dogs, the process of breed formation is likely to have not followed a bifurcating tree model and most breeds originated from crosses among multiple breeds. This is strongly supported by historical records that indicate that most modern rabbit breeds are the result of crossings between pre-existing varieties and outcrossing is often used for introgression of desirable characteristics in specific breeds, such as new coat colour mutants [10].

The lack of a deep hierarchical genetic structure in rabbits is also likely to be associated with a single domestication origin together with the short time elapsed between the initial domestication process and the rapid diversification of breeds in the last 200 years [10]. In some domestic animals, such as cattle, chicken, pigs or sheep, a hierarchically genetic structure comprised of genetic groups further subdivided in other groups can be observed, but the depth of this subdivision varies substantially and has distinct underlying causes [34,59,67–69]. The deeper substructure detected in many of these examples appears to result largely from genetic structure already present in the multiple wild populations or even species/subspecies that contributed to the domesticated gene pool, with pigs and cattle being two notorious examples [70,71]. Although less marked, genetic structure above the breed level has been also commonly associated with long periods of geographic isolation after domestication (*e*.*g*. sheep [59]) or between groups of breeds having distinct functional purposes (*e*.*g*. chicken [72,73]). It should be noted, however, that many relationships among breeds/varieties in all domesticated animals are characterized by unresolved polytomies similar to the overall pattern inferred for rabbits.

### Multiple causes underlying population structure within breeds

We detected additional levels of substructure within breeds using the clustering algorithm STRUCTURE (Figs 3 and S5). Interestingly, these clusters were in agreement with independent information regarding the sampled animals and we found three main sources of intrabreed structure. First, New Zealand rabbits that are composed of two distinct strains began to show a strong subdivision beyond *K* = 8. Second, the Rex breed, which consists of individuals belonging to three different colour morphs, began to show a subdivision signature between the strain Castor and the other two strains beyond *K* = 5, and later between Chinchilla and White strains (*K* = 25; S5 Fig). These clustering patterns suggest that genetic differentiation between these strains is stronger than that between many recognized breeds. Finally, the last source of breed subdivision was found for eight breeds and was associated with the breeder/country of origin. The evidence of intrabreed stratification documented here suggests that inbreeding practices, together with the isolation experienced by populations with different origins or different selection regimes, can work as strong barriers to gene flow and create genetic differentiation even between individuals of a single breed. Such an observation has previously been described for other domestic animals such as dogs [74] and sheep [75]. Population substructure has been demonstrated to potentially result in spurious phenotype/genotype associations in genome-wide association studies, highlighting the importance of a prior documentation of genetic structure [76]. Our results highlight the importance of taking into account the stratification within breeds when designing and interpreting genetic mapping studies in domestic rabbits.

## Supporting Information

S1 Fig Geographical location of the wild rabbit samples used in this study. The background layer reflects the elevation (increasing elevation from light to dark tones) highlighting the Pyrenees mountain range that separates the Iberian Peninsula from France.
(PDF)

S2 Fig Phylogenies of wild and domestic rabbits based on allele-sharing genetic distance. (A) Neighbour-joining tree for 471 individuals rooted with wild rabbits from the Iberian Peninsula. (B) Unrooted Neighbour-joining tree for 340 domestic individuals from 16 different breeds. Branches are coloured according to individual’s origin.
(PDF)

S3 Fig Average percentage of genetic diversity lost with different processes. Bars show values of genetic diversity lost for the colonization of France (green), domestication process (red), breed formation process (blue) and for each one of the 16 breeds (grey). Values were estimated using a resampling methodology (described in Methods). Error bars represent 95% confidence limit.
(PDF)

S4 Fig Consensus Fitch-Margoliash trees for 20 populations. Trees include domestic rabbits, wild French rabbits and is rooted with wild Iberian rabbits. (A) Tree based on chord genetic distance B) Tree based on allele-sharing genetic distance. Domestic rabbits include 16 breeds, two of which composed by two different strains. The node values correspond to the support values obtained from the consensus of 1000 trees and the branch length is proportional to support values.
(PDF)

S5 Fig STRUCTURE plots for different values of *K* for all 16 breeds. Five independent runs were performed for each *K* value, and *K* varied from 2 to 30.
(PDF)

S6 Fig Modal Distribution of ΔK for different values of *K* in STRUCTURE analysis with all domestic individuals. (PDF)

S7 Fig Discriminant Analysis of Principal Components (DAPC) of 16 breeds. The scatterplot shows the first two principal components (Y-axis and X-axis, respectively) using breeds as prior for genetic clusters. Each dot represents an individual, and genetic clusters are depicted by colours and 95% inertia ellipses. The eigenvalue components are show in the lower right pane with relative magnitude.
(PDF)

S1 File Microsatellite genotypes. The table contains individual rabbit genotypes for each microsatellite. Microsatellites are ordered according to the multiplex number order and group code represents breed/population.
(XLSX)

S1 Table Domestic breeds and strains with respective sample sizes. (PDF)

S2 Table Wild rabbit samples. (PDF)

S3 Table List of all 45 microsatellites used in this study. (PDF)

S4 Table Chromosome positions of the 12 microsatellites developed for this study. (PDF)

S5 Table Primers used for the 12 microsatellites developed for this study. (PDF)

S6 Table PCR profiles for the nine multiplex reactions. (PDF)

S7 Table Thermal cycler conditions for the nine multiplex PCRs. (PDF)

S8 Table Genetic diversity and sample sizes for each microsatellite. Values for domestic rabbits (DOM), wild rabbits from France (WF), and wild rabbits from the Iberian Peninsula (WIP).
(PDF)

S9 Table Pairwise F_ST_ genetic distances for all 16 domestic breeds. (PDF)

S10 Table Average percentage of genetic diversity lost during the colonization of France, domestication process, and the breed formation process. Values for breed formation process were averaged across breeds and for each individual breed. Values were estimated using a resampling methodology (described in the Methods section).
(PDF)

S11 Table Mean Similarity Coefficient for STRUCTURE runs from K = 2 to K = 9 (PDF)

S12 Table Chi-square test for Hardy-Weinberg equilibrium for all loci and across all 16 breeds (PDF)
