# Supplementary figures and images for: Levels and Patterns of Genetic Diversity and Population Structure in Domestic Rabbits
Source: PLoS One. 2015 Dec 21;10(12):e0144687. doi: 10.1371/journal.pone.0144687 (PMC4686922; doi:10.1371/journal.pone.0144687)

S1 Fig.

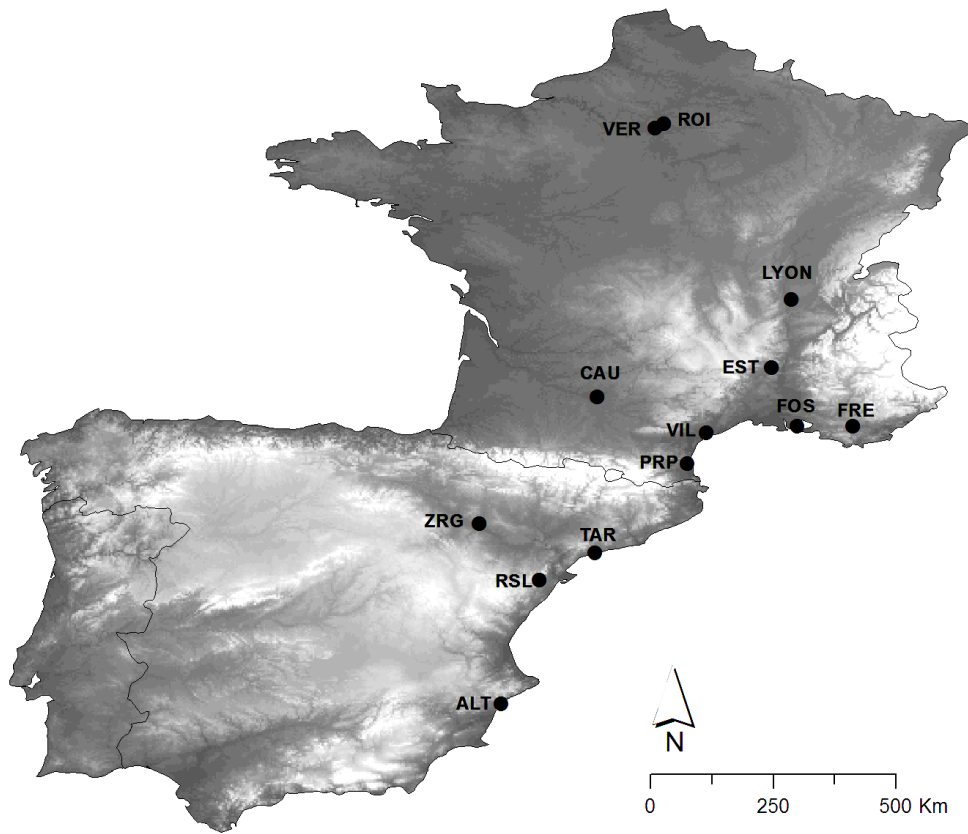

Supplement: S1 Fig — The background layer reflects the elevation (increasing elevation from light to dark tones) highlighting the Pyrenees mountain range that separates the Iberian Peninsula from France. (PDF) [file pone.0144687.s001.pdf]

S2 Fig.

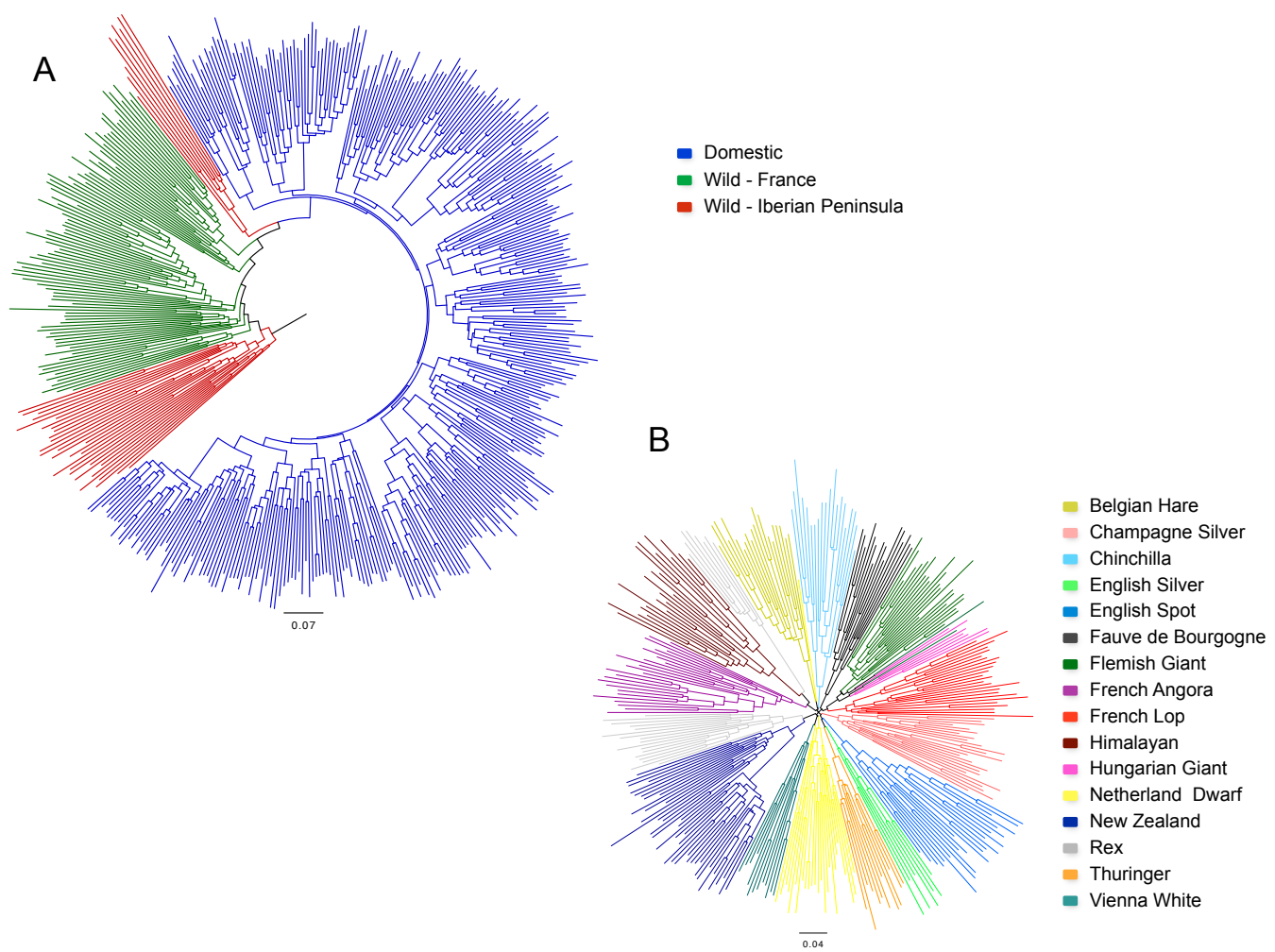

Supplement: S2 Fig — (A) Neighbour-joining tree for 471 individuals rooted with wild rabbits from the Iberian Peninsula. (B) Unrooted Neighbour-joining tree for 340 domestic individuals from 16 different breeds. Branches are coloured according to individual’s origin. (PDF) [file pone.0144687.s002.pdf]

S3 Fig.

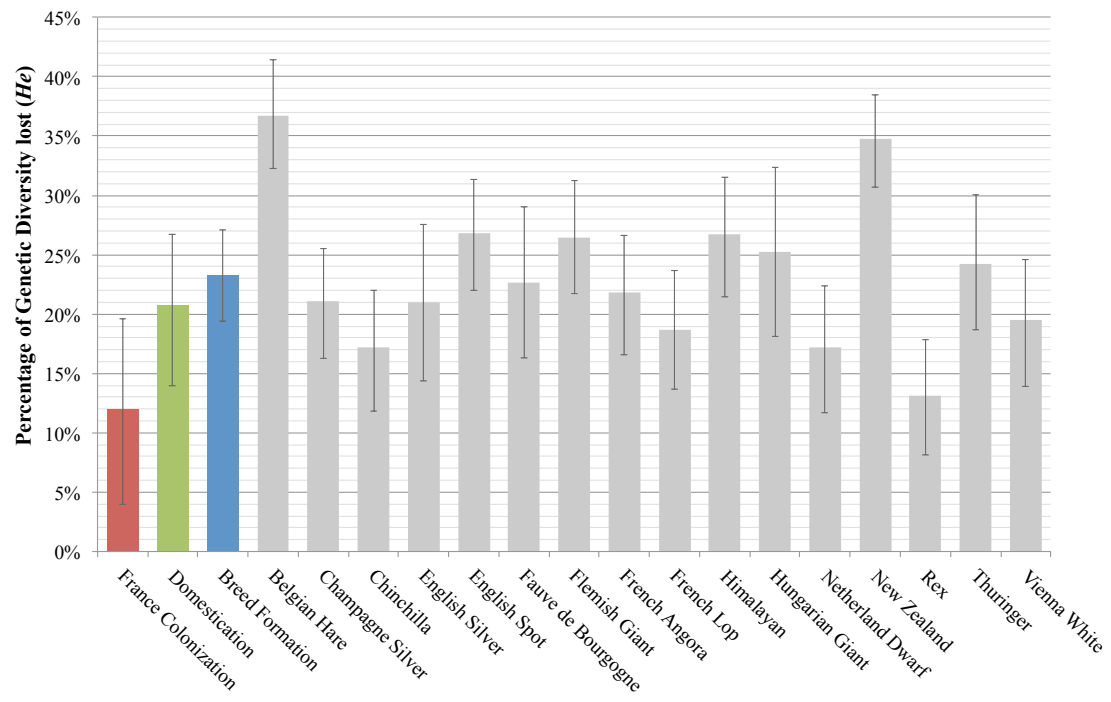

Supplement: S3 Fig — Bars show values of genetic diversity lost for the colonization of France (green), domestication process (red), breed formation process (blue) and for each one of the 16 breeds (grey). Values were estimated using a resampling methodology (described in Methods). Error bars represent 95% confidence limit. (PDF) [file pone.0144687.s003.pdf]

**S4 Fig.**

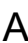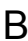

Supplement: S4 Fig — Trees include domestic rabbits, wild French rabbits and is rooted with wild Iberian rabbits. (A) Tree based on chord genetic distance B) Tree based on allele-sharing genetic distance. Domestic rabbits include 16 breeds, two of which composed by two different strains. The node values correspond to the support values obtained from the consensus of 1000 trees and the branch length is proportional to support values. (PDF) [file pone.0144687.s004.pdf]

S5 Fig.

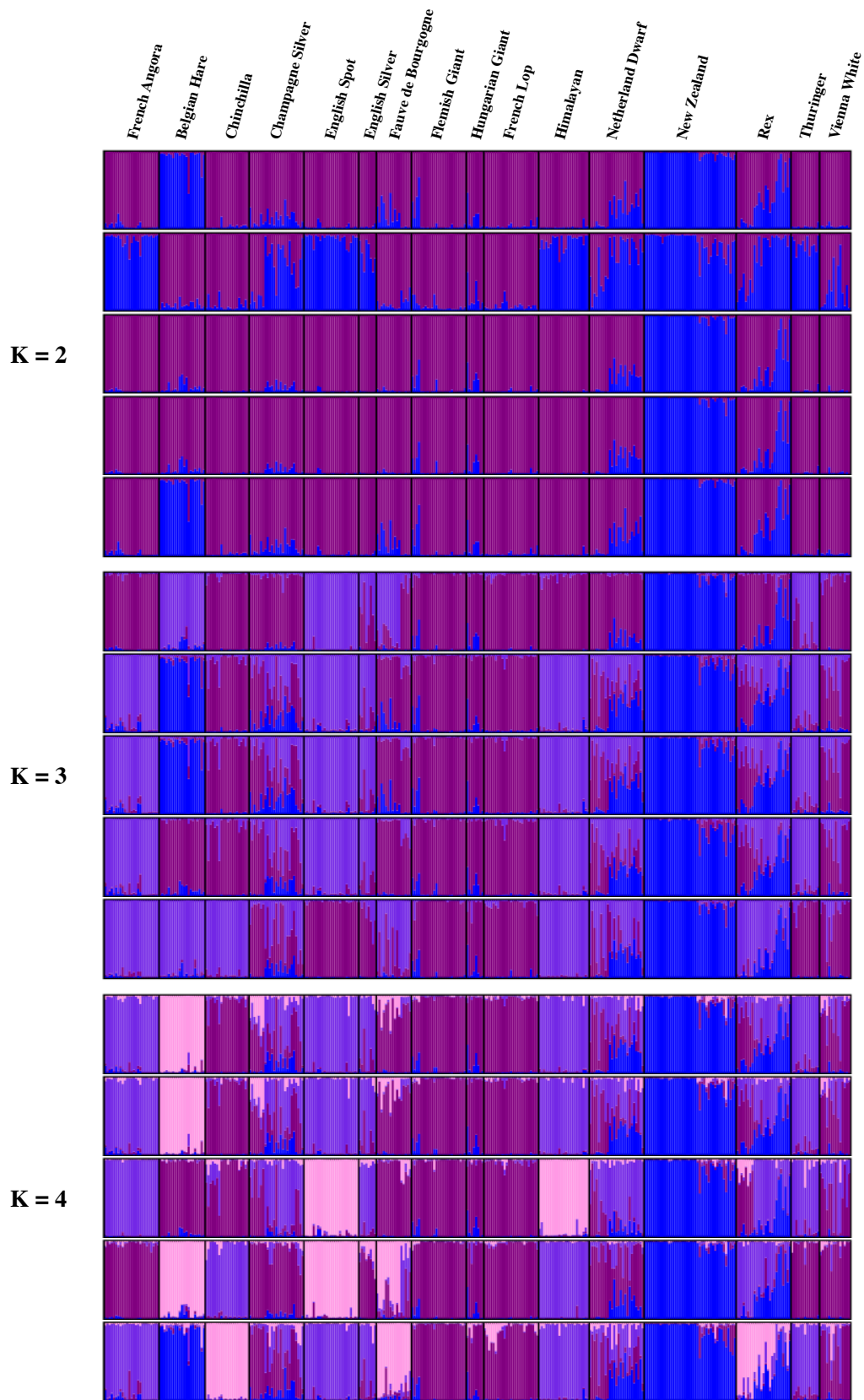

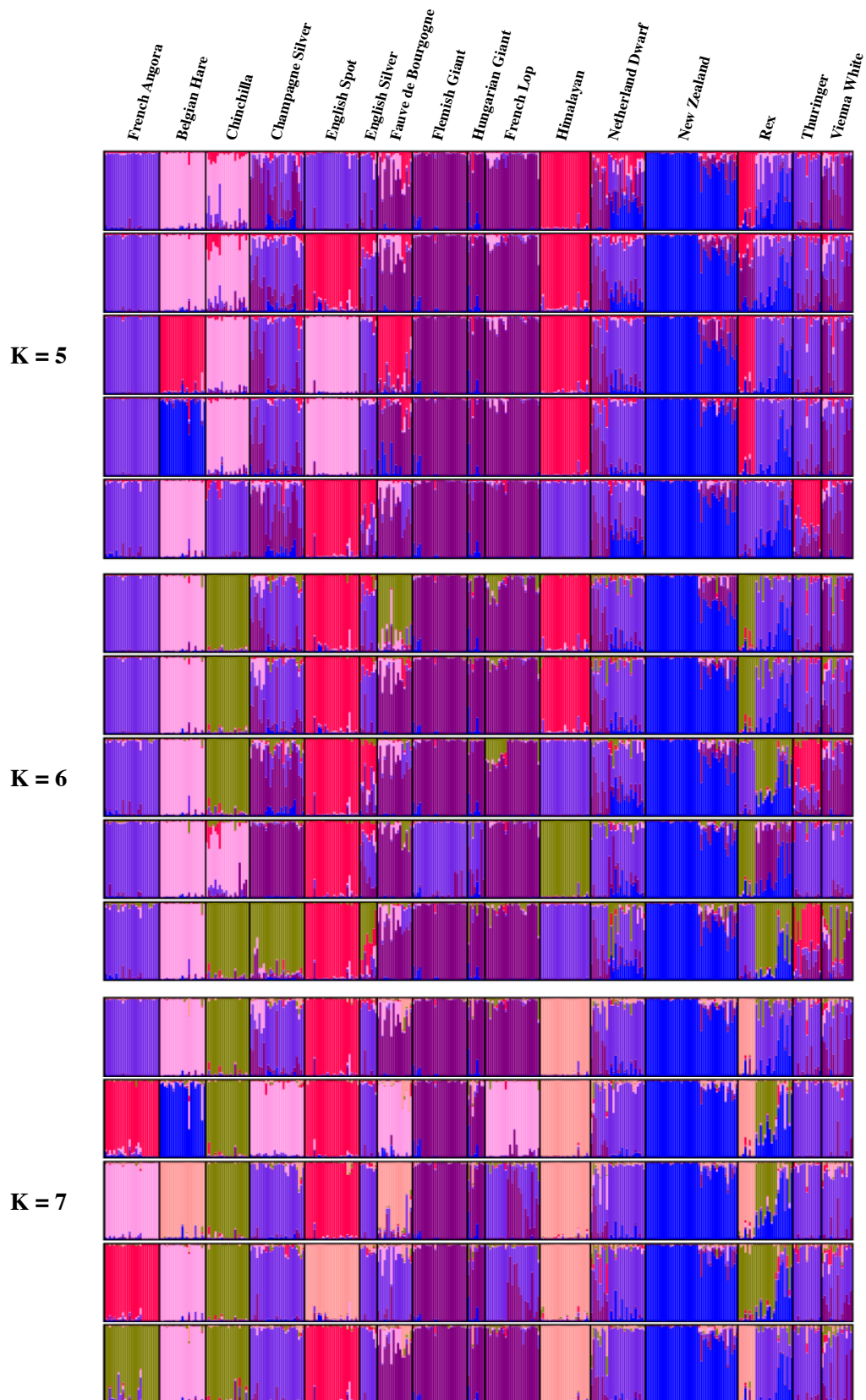

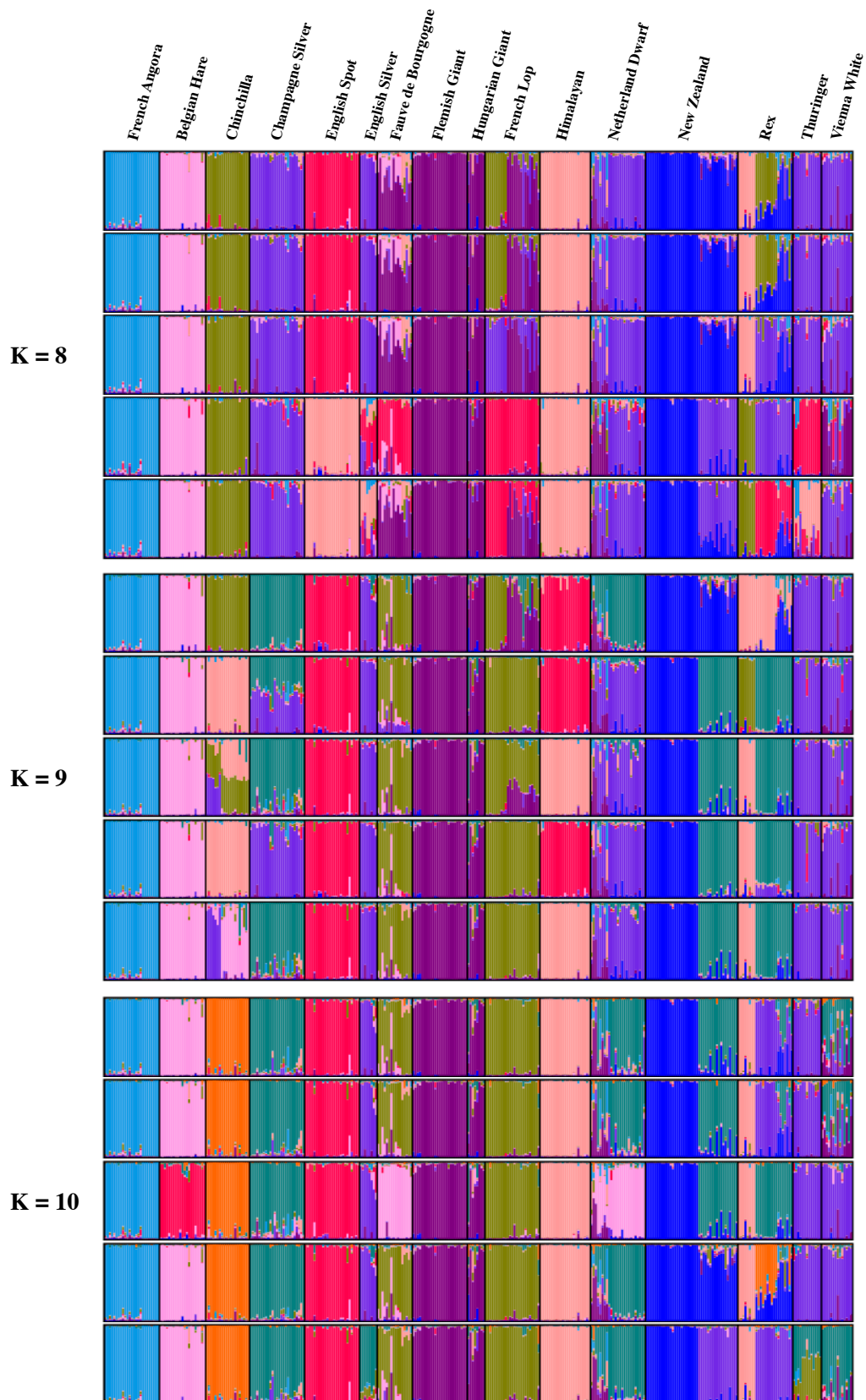

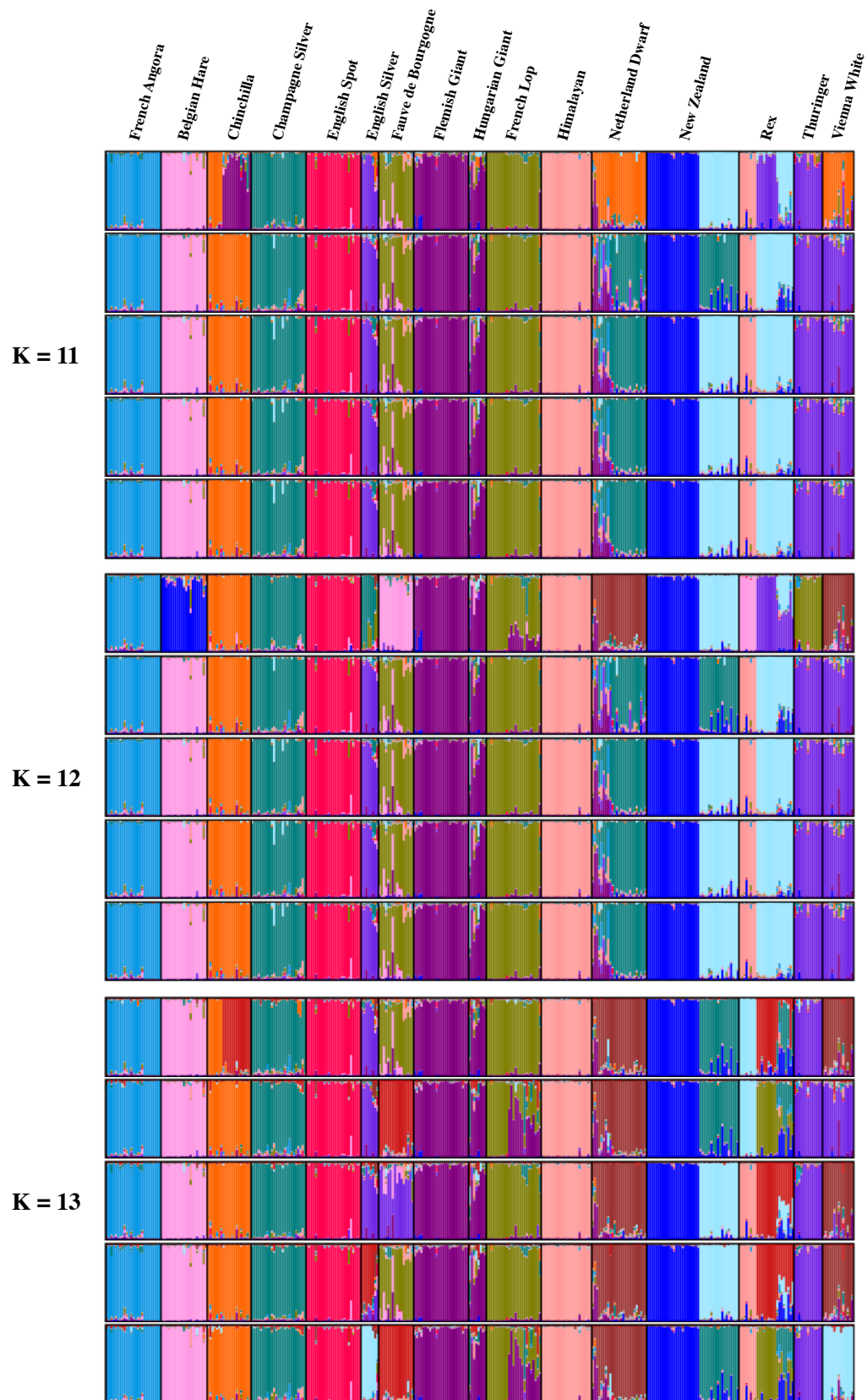

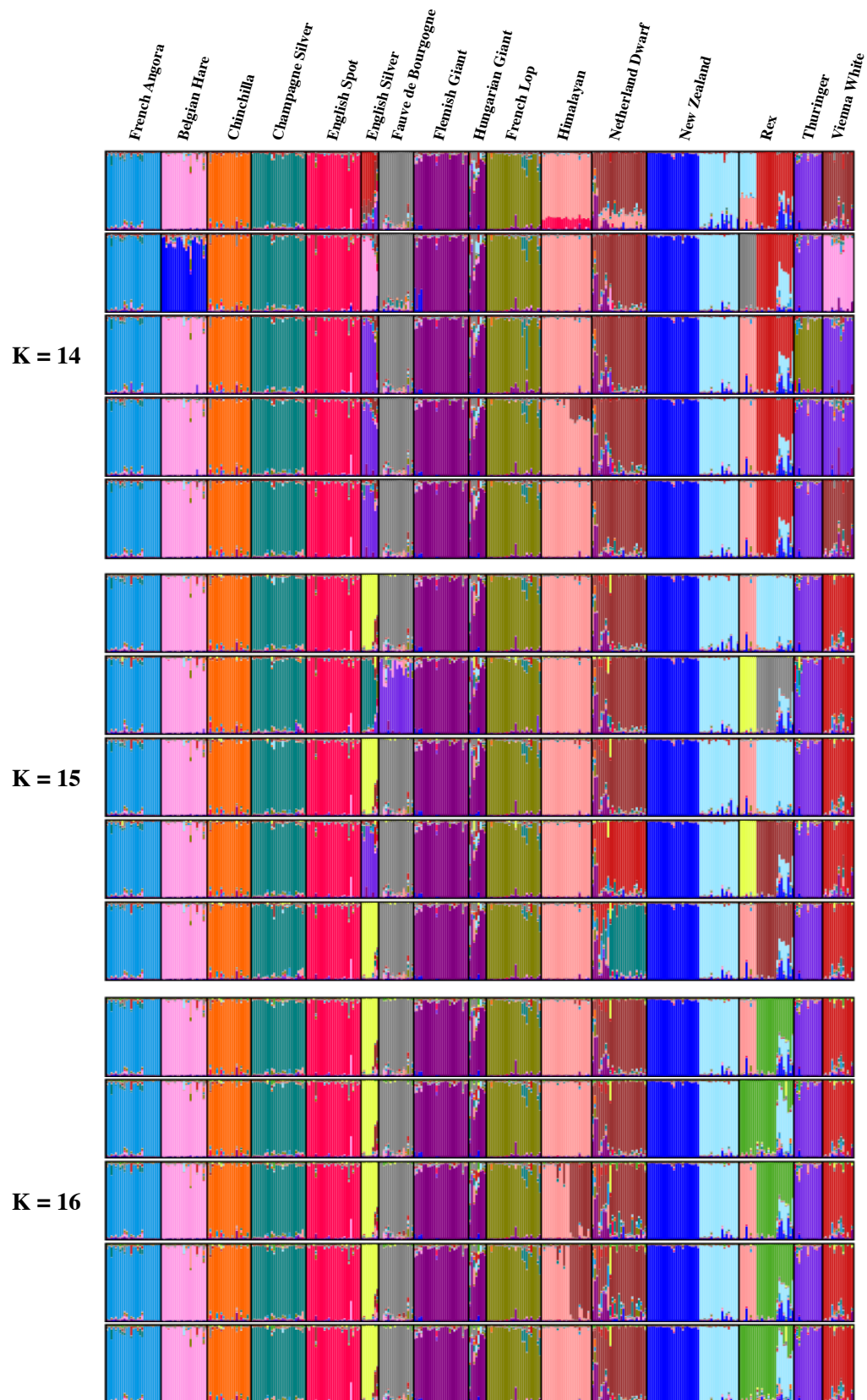



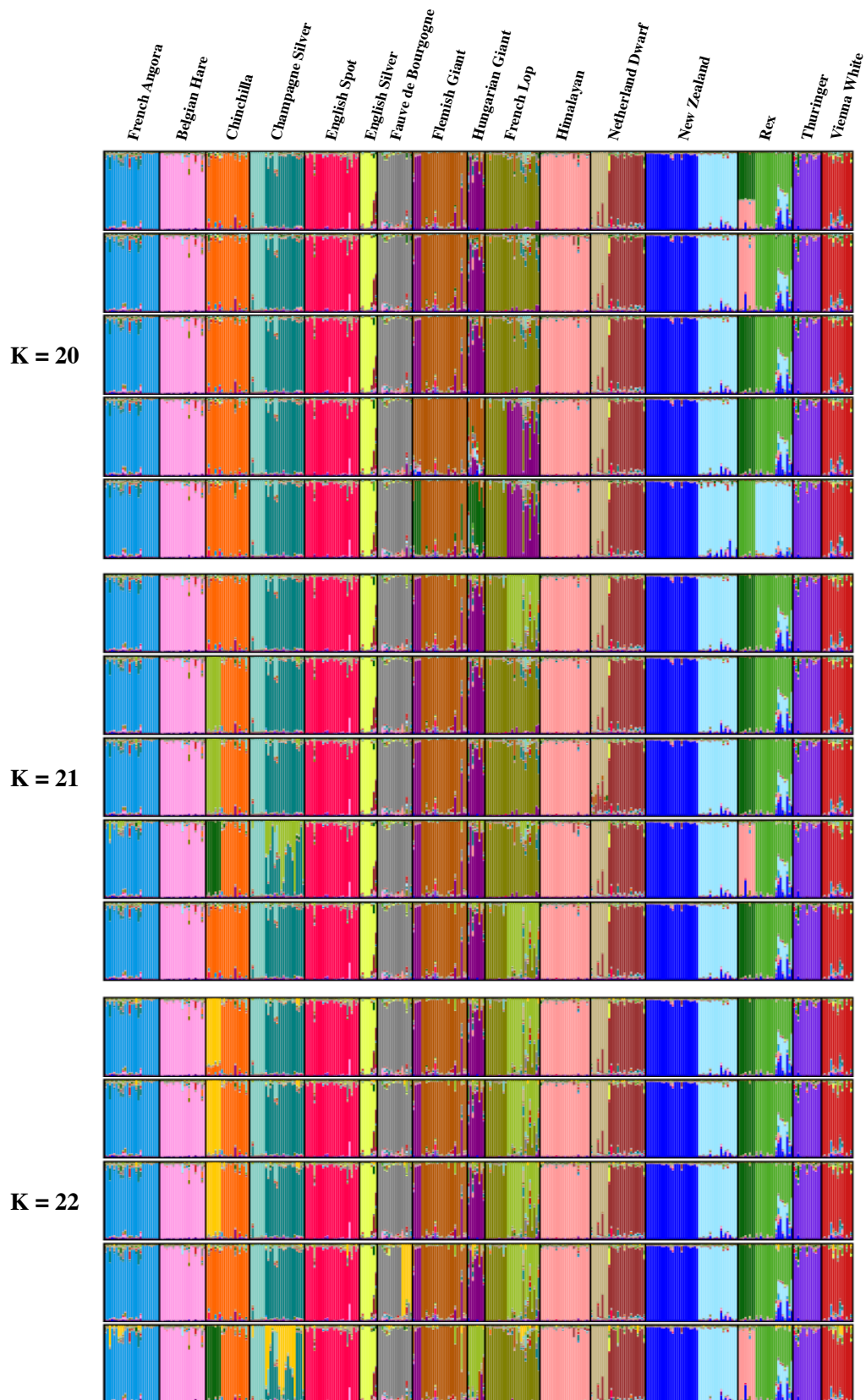

**K = 23**

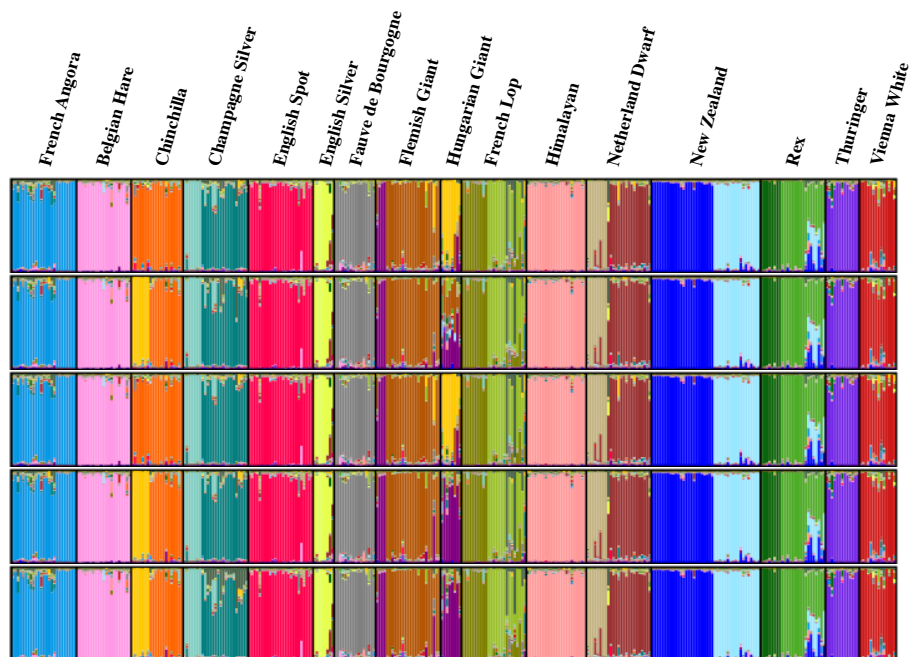

**K = 24**

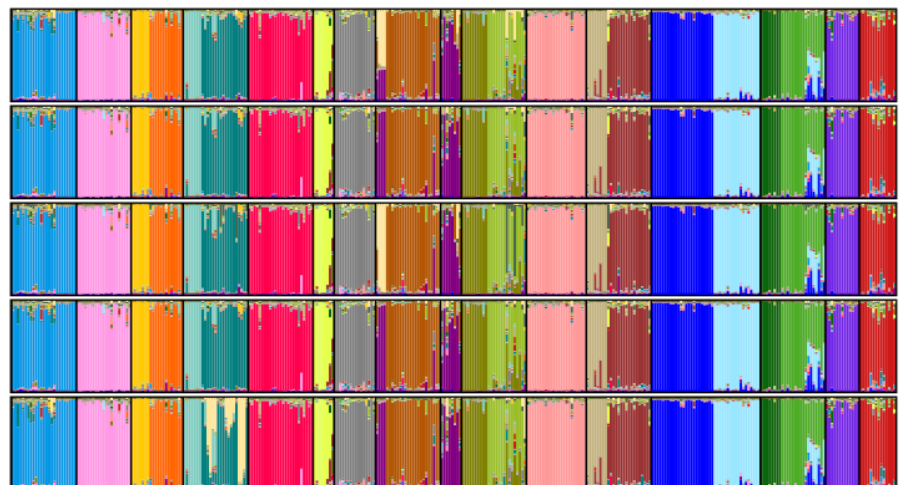

**K = 25**

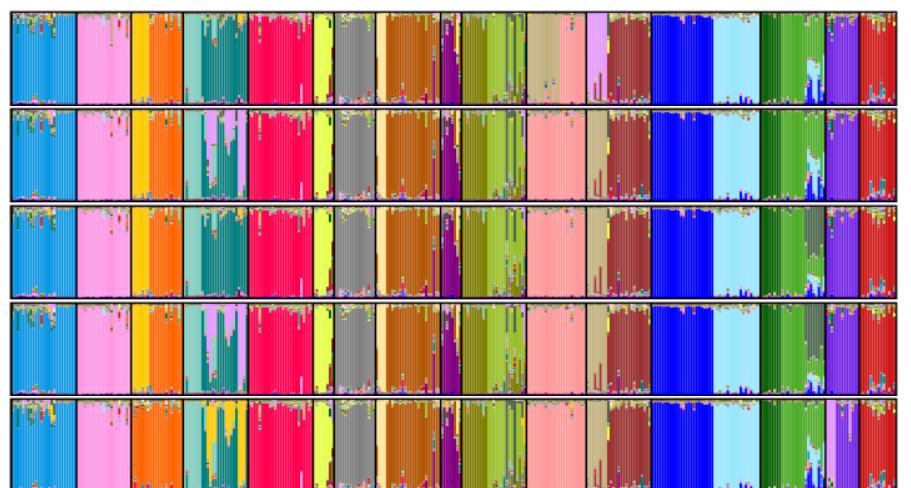

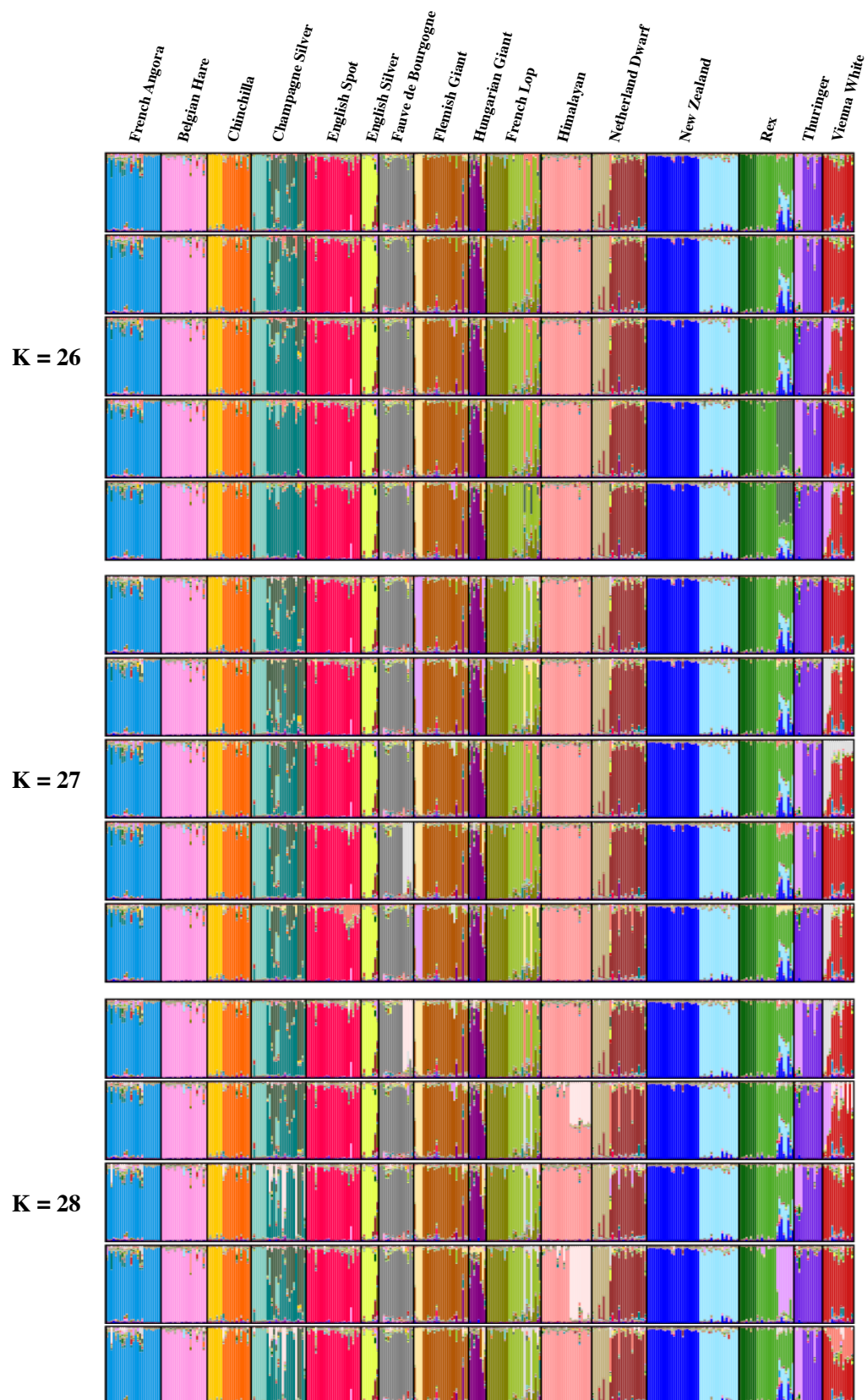

**K = 29**

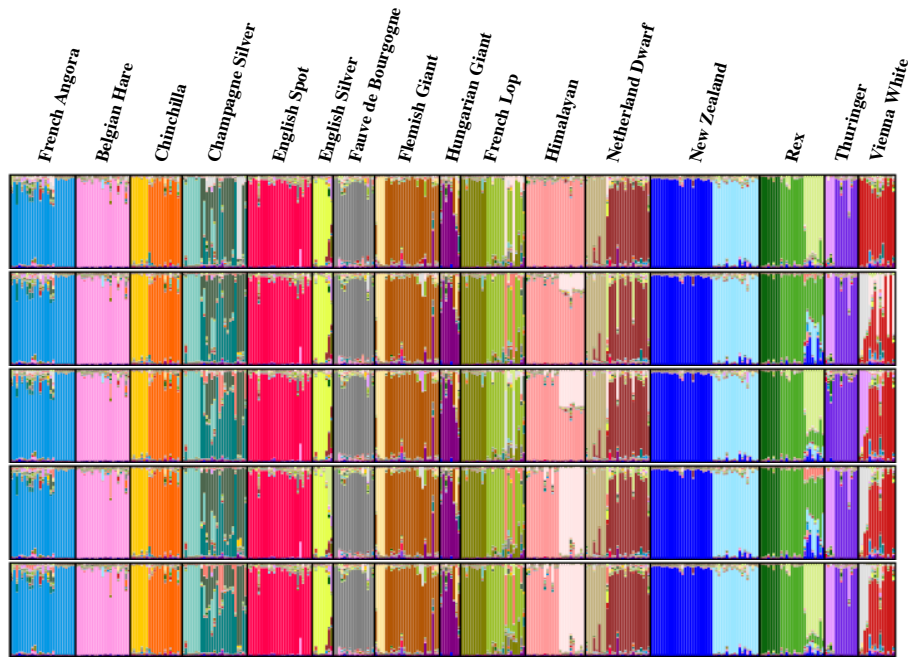

**K = 30**

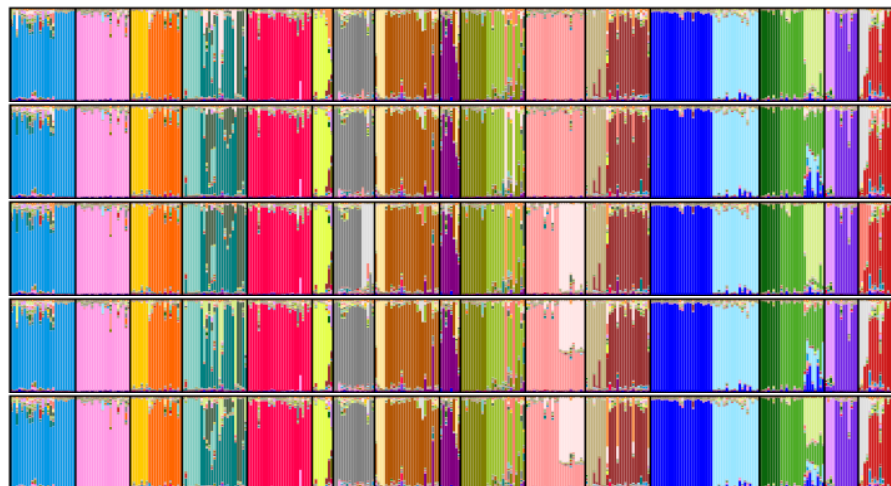

Supplement: S5 Fig — Five independent runs were performed for each K value, and K varied from 2 to 30. (PDF) [file pone.0144687.s005.pdf]

S6 Fig.

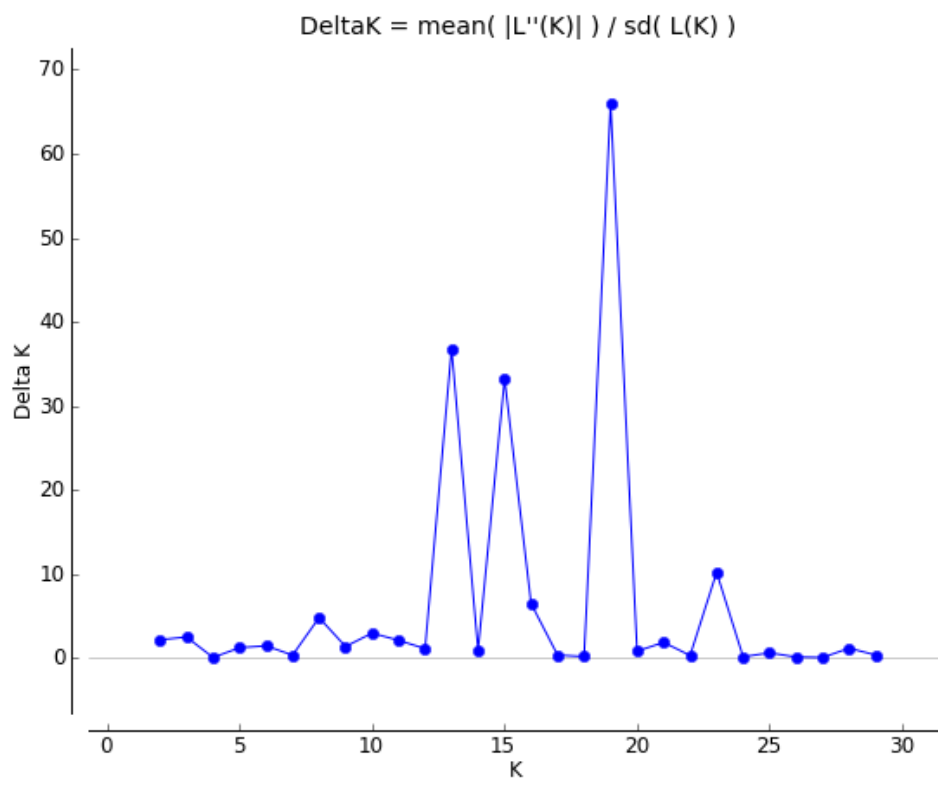

Supplement: S6 Fig — (PDF) [file pone.0144687.s006.pdf]

**S7 Fig.**

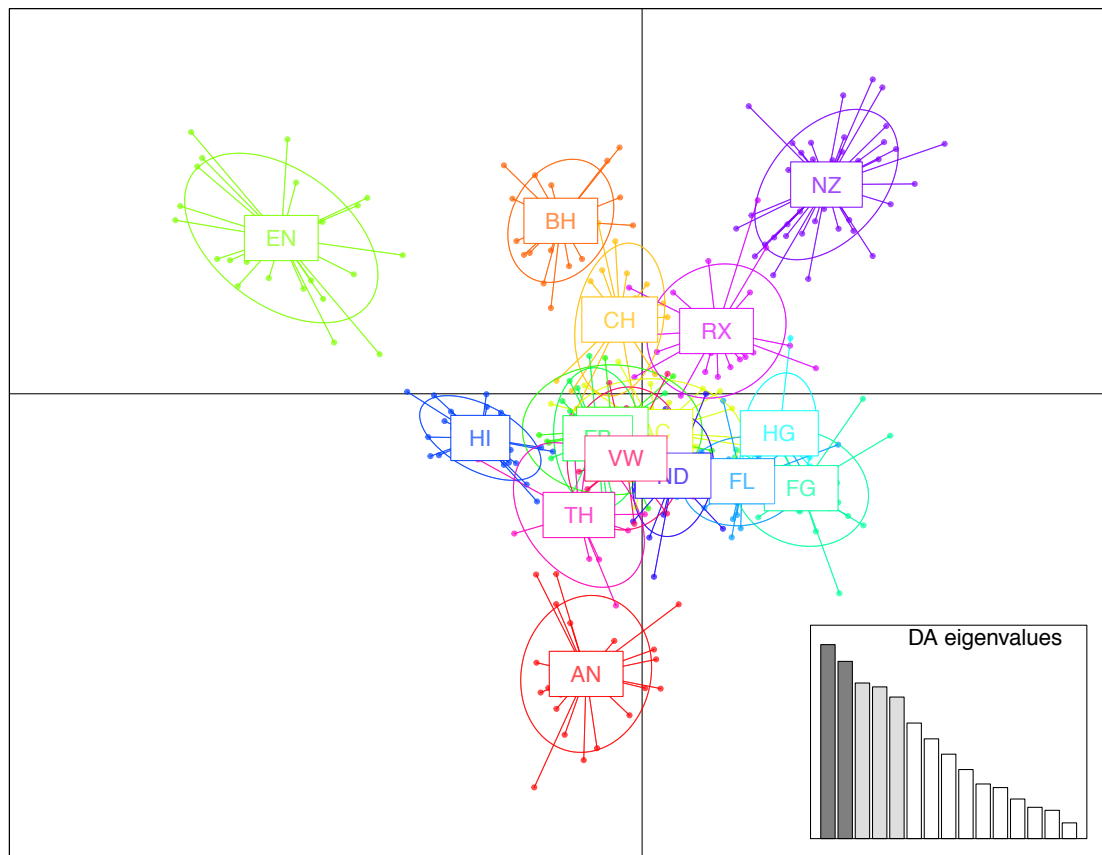

Supplement: S7 Fig — The scatterplot shows the first two principal components (Y-axis and X-axis, respectively) using breeds as prior for genetic clusters. Each dot represents an individual, and genetic clusters are depicted by colours and 95% inertia ellipses. The eigenvalue components are show in the lower right pane with relative magnitude. (PDF) [file pone.0144687.s007.pdf]
