# Supplementary material for: Levels and Patterns of Genetic Diversity and Population Structure in Domestic Rabbits
Source: PLoS One. 2015 Dec 21;10(12):e0144687. doi: 10.1371/journal.pone.0144687 (PMC4686922; doi:10.1371/journal.pone.0144687)
Supplement: S1 Table — (PDF) [file pone.0144687.s009.pdf]

S1 Table

**Breed**	**Strain**	**Code**	**Sample Size (*n*)**
Belgian Hare		BH	21
Champagne Silver		CS	25
Chinchilla		CH	20
English Silver		SL	8
English Spot		EN	25
Fauve de Bourgogne		FB	16
Flemish Giant		FG	25
French Angora		AN	25
French Lop		FL	25
Himalayan		HI	23
Hungarian Giant		HG	8
Netherland Dwarf		ND	25
New Zealand		NZ	42*
	INRA 9077	NZ_9077	18
	INRA 1077	NZ_1077	24
Rex		RX	17*
	Castor	RX_CA	9
	Chinchilla	RX_CH	9
	White	RX_WH	8
Thuringer		TH	13
Vienna White		VW	14

*Total number of individuals belonging to the different strains
