# Supplementary material for: Levels and Patterns of Genetic Diversity and Population Structure in Domestic Rabbits
Source: PLoS One. 2015 Dec 21;10(12):e0144687. doi: 10.1371/journal.pone.0144687 (PMC4686922; doi:10.1371/journal.pone.0144687)
Supplement: S2 Table — (PDF) [file pone.0144687.s010.pdf]

S2 Table

**Region**	**Localities**	**Code**[a]	**Sample Size (*n*)**
France (*n*=92)	Causse	CAU	11
	Estagnol	EST	4
	Fos sur Mer	FOS	4
	Frejus	FRE	4
	Lyon	LYO	27
	Perpignan	PRP	10
	Roissy	ROI	10
	Versailles	VER	19
	Villeneuve	VIL	3
Iberian Peninsula (*n*=39)	Alicante	ALT	8
	Rosell	RSL	9
	Tarragona	TAR	9
	Zaragoza	ZRG	13

[a]Locality codes are present in Supplementary Figure 1
