# Supplementary material for: Levels and Patterns of Genetic Diversity and Population Structure in Domestic Rabbits
Source: PLoS One. 2015 Dec 21;10(12):e0144687. doi: 10.1371/journal.pone.0144687 (PMC4686922; doi:10.1371/journal.pone.0144687)
Supplement: S3 Table — (PDF) [file pone.0144687.s011.pdf]

S3 Table

Marker	Original Code	Chromosome	Repeat Pattern	Product Size	Accession Nº	Reference
STR01	SAT03	3	$(TC)_{22}$	146-162	J03744	Mougel et al. 1997
STR02	SAT04	15	$(TC)_{13}(N)_{5}(TC)_{2}TG(TC)_{7}$	195-240	M33582	Mougel et al. 1997
STR03	SAT05	3	$(TC)_{23}TTT(CT)_{5}$	206-234	X99887	Mougel et al. 1997
STR04	SAT07	10	$(TG)_{14}$	184-195	X99888	Mougel et al. 1997
STR05	SAT08	17	$(CT)_{14}(GT)_{8}TT(GT)_{5}$	136-158	X99889	Mougel et al. 1997
STR06	SAT12	7	$(CTAT)_{10}$	122-138	X99891	Mougel et al. 1997
STR07	SAT13	1	$(GT)_{13}$	114-128	X99892	Mougel et al. 1997
STR08	INRACCDDV0040	4	$(TG)_{16}$	197	AJ874400	Chantry-Darmon et al. 2005
STR09	INRACCDDV0087	8	$(TG)_{14}$	194	AJ874430	Chantry-Darmon et al. 2005
STR10	INRACCDDV0089	19	$(CA)_{14}$	80	AJ874432	Chantry-Darmon et al. 2005
STR11	INRACCDDV0101	11	$(TG)_{12}$	140	AJ874443	Chantry-Darmon et al. 2005
STR12	INRACCDDV0102	19	$(AC)_{18}$	219	AJ874444	Chantry-Darmon et al. 2005
STR13	INRACCDDV0104	18	$(GT)_{15}$	108	AJ874446	Chantry-Darmon et al. 2005
STR14	INRACCDDV0106	13	$(CA)_{14}$	156	AJ874448	Chantry-Darmon et al. 2005
STR15	INRACCDDV0108	11	$(CA)_{13}$	130	AJ874450	Chantry-Darmon et al. 2005
STR16	INRACCDDV0119	18	$(GT)_{16}$	210	AJ874461	Chantry-Darmon et al. 2005
STR17	INRACCDDV0139	13	$(TG)_{16}$	116	AJ874479	Chantry-Darmon et al. 2005
STR18	INRACCDDV0140	14	$(TG)_{14}$	165	AJ874480	Chantry-Darmon et al. 2005
STR19	INRACCDDV0157	8	$(GT)_{12}$	120	AJ874497	Chantry-Darmon et al. 2005
STR20	INRACCDDV0169	1	$(CA)_{17}$	151	AJ874508	Chantry-Darmon et al. 2005
STR21	INRACCDDV0172	17	$(AC)_{14}$	86	AJ874510	Chantry-Darmon et al. 2005
STR22	INRACCDDV0176	12	$(TC)_{11}(TG)_{13}AG(TG)_{3}$	208	AJ874514	Chantry-Darmon et al. 2005
STR23	INRACCDDV0182	4	$(TG)_{20}$	93	AJ874520	Chantry-Darmon et al. 2005
STR24	INRACCDDV0185	16	$(AC)_{13}$	155	AJ874523	Chantry-Darmon et al. 2005
STR25	INRACCDDV0190	18	$(TG)_{12}$	189	AJ874528	Chantry-Darmon et al. 2005
STR26	INRACCDDV0192	2	$(TG)_{11}$	96	AJ874530	Chantry-Darmon et al. 2005
STR27	INRACCDDV0201	12	$(TG)_{14}(AG)_{10}$	117	AJ874538	Chantry-Darmon et al. 2005
STR28	INRACCDDV0203	3	$(GT)_{16}$	184	AJ874540	Chantry-Darmon et al. 2005

STR29	INRACCDDV0205	4	$(TG)_{17}$	170	AJ874542	Chantry-Darmon et al. 2005
STR30	INRACCDDV0228	-	$(TG)_{12}$	217	AJ874561	Chantry-Darmon et al. 2005
STR31	INRACCDDV0259	1	$(GT)_{14}(GA)_{9}$	153	AJ874589	Chantry-Darmon et al. 2005
STR32	INRACCDDV0313	14	$(TC)_{7}(AC)_{10}GC(AC)_{6}GC(AC)_{8}$	235	AJ874634	Chantry-Darmon et al. 2005
STR33	INRACCDDV0342	13	$(GA)_{22}$	170	AJ874659	Chantry-Darmon et al. 2005
STR34	-	X	$(AG)_{12}$	186	-	This Study
STR35	-	X	$(CA)_{10}(GA)_{11}$	236	-	This Study
STR36	-	8	$(CT)_{19}$	186	-	This Study
STR37	-	4	$(CA)_{13}$	224	-	This Study
STR38	-	15	$(GT)_{18}$	227	-	This Study
STR39	-	3	$(GTCT)3(CT)_{8}$	131	-	This Study
STR40	-	X	$(GA)_{16}$	204	-	This Study
STR41	-	X	$(GT)_{10}$	109	-	This Study
STR42	-	X	$(GA)_{9}$	228	-	This Study
STR43	-	X	$(GA)_{15}$	137	-	This Study
STR44	-	X	$(AC)_{13}$	240	-	This Study
STR45	-	7	$(AG)_{18}$	112	-	This Study
