# Supplementary material for: Levels and Patterns of Genetic Diversity and Population Structure in Domestic Rabbits
Source: PLoS One. 2015 Dec 21;10(12):e0144687. doi: 10.1371/journal.pone.0144687 (PMC4686922; doi:10.1371/journal.pone.0144687)
Supplement: S4 Table — (PDF) [file pone.0144687.s012.pdf]

S4 Table

Marker	Chromosome	Position (Mb)
STR34	X	61.4
STR35	X	42.3
STR36	8	44.1
STR37	4	13.4
STR38	15	17.1
STR39	3	50.1
STR40	X	Unknown
STR41	X	4.9
STR42	X	97.9
STR43	X	108.9
STR44	X	66.6
STR45	7	59.2
