# Supplementary material for: Levels and Patterns of Genetic Diversity and Population Structure in Domestic Rabbits
Source: PLoS One. 2015 Dec 21;10(12):e0144687. doi: 10.1371/journal.pone.0144687 (PMC4686922; doi:10.1371/journal.pone.0144687)
Supplement: S5 Table — (PDF) [file pone.0144687.s013.pdf]

S5 Table

Marker	Primer Forward	Primer Reverse
STR34	5’ CAAAGCAAATACAAGGCTTC 3’	5’ GAGAGTGAGCCAGTGGATAG 3’
STR35	5’ TCTTTCCCATGTATTGCAG 3’	5’ ATGTGGCCCAATTCCTAT 3’
STR36	5’ GGAGACCTGGATGGAGTT 3’	5’ GAGAGAGGGGCATCACTT 3’
STR37	5’ CTCACCACTTCAGGTTTGAT 3’	5’ TTGTAGATACCTGGGGAGTG 3’
STR38	5’ GTAACTGCTGTGGCATCC 3’	5’ GCAGTGACCCTTCCTTC 3’
STR39	5’ GCCATTTGGGAAGTGAAT 3’	5’ CCTGTGCTGCTAGTGTCC 3’
STR40	5’ ACAGAGAGAGGAGGAGAGC 3’	5’ TGGCCTAGGAAAGCATTA 3’
STR41	5’ GTGCCCAGCTGAGTATTG 3’	5’ GGACCCTGTGATTTAGGG 3’
STR42	5’ GCCTGTCCAGTTCATACAG 3’	5’ CAGAAGGTGATGGGTCAA 3’
STR43	5’ CTGATGCAATGGTCCTGT 3’	5’ TTGGGGAGTGAACTAGCA 3’
STR44	5’ CAGTGTTTGTCAGTCACCTC 3’	5’ TGGCACAGGAATAGGTTG 3’
STR45	5’ CCCCTCCCACACTACTCT 3’	5’ CATCTGGGGGAGTTGAA 3’
