# Supplementary material for: Levels and Patterns of Genetic Diversity and Population Structure in Domestic Rabbits
Source: PLoS One. 2015 Dec 21;10(12):e0144687. doi: 10.1371/journal.pone.0144687 (PMC4686922; doi:10.1371/journal.pone.0144687)
Supplement: S6 Table — (PDF) [file pone.0144687.s014.pdf]

# S6 Table

| **Multiplex 1** | | **Multiplex 2** | | **Multiplex 3** | | **Multiplex 4** | | **Multiplex 5** | |
|---|---|---|---|---|---|---|---|---|---|
| **Marker** | **Dye** | **Marker** | **Dye** | **Marker** | **Dye** | **Marker** | **Dye** | **Marker** | **Dye** |
| STR15 | FAM | STR10 | FAM | STR01 | FAM | STR12 | FAM | STR32 | FAM |
| STR21 | FAM | STR18 | FAM | STR02 | FAM | STR07 | NED | STR23 | NED |
| STR22 | FAM | STR09 | NED | STR03 | NED | STR20 | NED | STR08 | PET |
| STR14 | NED | STR19 | NED | STR05 | PET | STR13 | PET | STR24 | PET |
| STR17 | NED | STR25 | PET | STR04 | VIC | STR29 | PET | STR31 | VIC |
| STR11 | PET | STR06 | PET | | | STR30 | PET | | |
| STR28 | PET | STR16 | VIC | | | STR26 | VIC | | |
| | | STR27 | VIC | | | | | | |

| **Multiplex 6** | | **Multiplex 7** | | **Multiplex 8** | | **Multiplex 9** | |
|---|---|---|---|---|---|---|---|
| **Marker** | **Dye** | **Marker** | **Dye** | **Marker** | **Dye** | **Marker** | **Dye** |
| STR44 | FAM | STR37 | FAM | STR42 | NED | STR40 | NED |
| STR45 | FAM | STR35 | NED | STR34 | VIC | STR36 | VIC |
| STR38 | VIC | STR43 | PET | STR39 | | | |
| STR41 | VIC | STR33 | VIC | | | | |
