# Supplementary material for: Levels and Patterns of Genetic Diversity and Population Structure in Domestic Rabbits
Source: PLoS One. 2015 Dec 21;10(12):e0144687. doi: 10.1371/journal.pone.0144687 (PMC4686922; doi:10.1371/journal.pone.0144687)
Supplement: S7 Table — (PDF) [file pone.0144687.s015.pdf]

S7 Table

**Multiplex 1**			**Multiplex 2**			**Multiplex 3**			**Multiplex 4**			**Multiplex 5**		
**1x**	95 ^{0}C	15 min	**1x**	95 ^{0}C	15 min	**1x**	95 ^{0}C	15 min	**1x**	95 ^{0}C	15 min	**1x**	95 ^{0}C	15 min
**16x**	95 ^{0}C	30 s	**12x**	95 ^{0}C	30 s	**38x**	95 ^{0}C	30 s	**10x**	95 ^{0}C	30 s	**14x**	95 ^{0}C	30 s
	63 - 55^{0}C (↓0,5^{0}C)	30 s		61 - 55^{0}C (↓0,5^{0}C)	45 s		60 ^{0}C	30 s		59 - 54^{0}C (↓0,5^{0}C)	30 s		63 - 56^{0}C (↓0,5^{0}C)	30 s
	72 ^{0}C	45 s		72 ^{0}C	45 s		72 ^{0}C	45 s		72 ^{0}C	45 s		72 ^{0}C	45 s
**25x**	95 ^{0}C	30 s	**25x**	95 ^{0}C	30 s	**7x**	95 ^{0}C	30 s	**25x**	95 ^{0}C	30 s	**25x**	95 ^{0}C	30 s
	55 ^{0}C	30 s		55 ^{0}C	45 s		53 ^{0}C	30 s		54 ^{0}C	30 s		56 ^{0}C	30 s
	72 ^{0}C	45 s		72 ^{0}C	45 s		72 ^{0}C	45 s		72 ^{0}C	45 s		72 ^{0}C	45 s
**7x**	95 ^{0}C	30 s	**7x**	95 ^{0}C	30 s	**1x**	60 ^{0}C	20 min	**7x**	95 ^{0}C	30 s	**7x**	95 ^{0}C	30 s
	53 ^{0}C	30 s		53 ^{0}C	45 s					53 ^{0}C	30 s		53 ^{0}C	30 s
	72 ^{0}C	45 s		72 ^{0}C	45 s					72 ^{0}C	45 s		72 ^{0}C	45 s
**1x**	60 ^{0}C	20 min	**1x**	60 ^{0}C	20 min				**1x**	60 ^{0}C	20 min	**1x**	60 ^{0}C	20 min

**Multiplex 6**			**Multiplex 7**			**Multiplex 8**			**Multiplex 9**		
**1x**	95 ^{0}C	15 min	**1x**	95 ^{0}C	15 min	**1x**	95 ^{0}C	15 min	**1x**	95 ^{0}C	15 min
**8x**	95 ^{0}C	30 s	**14x**	95 ^{0}C	30 s	**10x**	95 ^{0}C	30 s	**16x**	95 ^{0}C	30 s
	66 - 62^{0}C (↓0,5^{0}C)	30 s		67 - 60^{0}C (↓0,5^{0}C)	30 s		65 - 60^{0}C (↓0,5^{0}C)	30 s		67 - 59^{0}C (↓0,5^{0}C)	30 s
	72 ^{0}C	45 s		72 ^{0}C	45 s		72 ^{0}C	45 s		72 ^{0}C	45 s
**25x**	95 ^{0}C	30 s	**25x**	95 ^{0}C	30 s	**25x**	95 ^{0}C	30 s	**25x**	95 ^{0}C	30 s
	62 ^{0}C	30 s		60 ^{0}C	30 s		60 ^{0}C	30 s		59 ^{0}C	30 s
	72 ^{0}C	45 s		72 ^{0}C	45 s		72 ^{0}C	45 s		72 ^{0}C	45 s
**7x**	95 ^{0}C	30 s	**7x**	95 ^{0}C	30 s	**7x**	95 ^{0}C	30 s	**7x**	95 ^{0}C	30 s
	53 ^{0}C	30 s		53 ^{0}C	30 s		53 ^{0}C	30 s		53 ^{0}C	30 s
	72 ^{0}C	45 s		72 ^{0}C	45 s		72 ^{0}C	45 s		72 ^{0}C	45 s
**1x**	60 ^{0}C	20 min	**1x**	60 ^{0}C	20 min	**1x**	60 ^{0}C	20 min	**1x**	60 ^{0}C	20 min

The symbol ↓ represents a touchdown step, in which the temperature was decreased by 0,5^{0}C in each cycle.
