# Supplementary material for: Levels and Patterns of Genetic Diversity and Population Structure in Domestic Rabbits
Source: PLoS One. 2015 Dec 21;10(12):e0144687. doi: 10.1371/journal.pone.0144687 (PMC4686922; doi:10.1371/journal.pone.0144687)
Supplement: S8 Table — Values for domestic rabbits (DOM), wild rabbits from France (WF), and wild rabbits from the Iberian Peninsula (WIP). (PDF) [file pone.0144687.s016.pdf]

S8 Table

Locus	DOM						WF						WIP					
	n	*Na*	*Ar*	*PAr*	*He*	*F*	*n*	*Na*	*Ar*	*PAr*	*He*	*F*	*n*	*Na*	*Ar*	*PAr*	*He*	*F*
STR01	332	9	5.253	0.387	0.674	0.203	90	11	9.526	3.416	0.811	0.132	39	15	14.303	12.260	0.915	0.120
STR02	309	11	6.612	0.405	0.762	0.396	88	15	10.741	1.817	0.802	0.231	39	16	15.051	5.743	0.903	0.051
STR03	306	9	5.302	0.052	0.521	0.453	87	11	8.331	0.634	0.768	0.112	39	18	16.862	9.008	0.914	-0.023
STR04	330	7	5.392	0.179	0.752	0.427	90	10	7.961	0.345	0.803	0.068	39	10	9.786	2.371	0.876	0.022
STR05	332	4	3.093	0.019	0.621	0.354	91	8	5.242	0.444	0.557	0.147	39	17	15.719	10.848	0.837	0.069
STR06	276	6	4.772	0.072	0.713	0.659	80	7	5.982	0.948	0.742	0.525	35	5	4.988	1.001	0.660	0.693
STR07	335	6	5.147	0.050	0.477	0.273	92	7	6.221	0.277	0.775	0.055	39	11	10.225	3.146	0.754	0.139
STR08	338	6	5.159	0.035	0.665	0.322	87	10	7.867	1.128	0.805	0.024	39	11	10.541	2.879	0.862	0.217
STR09	318	10	9.563	1.057	0.852	0.291	88	10	8.716	0.010	0.852	0.048	39	9	8.786	1.001	0.863	0.067
STR10	330	5	3.857	0.036	0.367	0.479	81	5	3.623	0.562	0.243	0.081	39	4	4.000	0.103	0.691	0.173
STR11	324	5	3.707	0.000	0.408	0.249	87	8	6.484	0.016	0.693	0.283	38	11	10.778	4.300	0.868	0.447
STR12	338	5	4.922	0.001	0.729	0.232	90	11	9.417	1.594	0.836	-0.016	39	15	13.847	7.011	0.882	0.057
STR13	338	6	5.161	0.018	0.588	0.355	92	6	4.994	0.000	0.637	0.143	39	8	7.960	2.260	0.851	0.145
STR14	314	6	5.117	0.000	0.712	0.369	89	7	6.992	0.040	0.844	0.210	39	10	9.550	2.590	0.859	0.486
STR15	307	6	3.739	0.000	0.659	0.252	87	7	6.154	0.356	0.652	0.096	38	7	6.995	1.122	0.819	0.251
STR16	329	5	3.772	0.180	0.578	0.474	86	7	6.707	0.008	0.779	0.279	39	11	10.983	4.223	0.903	0.252
STR17	326	5	4.948	0.001	0.716	0.305	89	6	5.897	0.199	0.746	0.121	39	7	6.748	0.988	0.741	0.054
STR18	329	5	3.358	0.360	0.532	0.211	87	4	3.519	0.000	0.545	0.194	39	7	6.753	2.822	0.698	0.182
STR19	280	7	4.705	0.599	0.728	0.587	85	6	5.558	0.879	0.645	0.285	39	8	7.755	3.181	0.807	0.292
STR20	338	7	5.123	0.401	0.717	0.319	92	8	7.052	0.049	0.745	0.061	39	9	8.549	2.059	0.788	0.175
STR21	300	4	3.422	0.000	0.620	0.317	80	5	4.387	0.000	0.689	0.489	31	8	8.000	3.354	0.788	0.335
STR22	325	6	3.640	0.333	0.244	0.810	78	18	14.068	1.864	0.743	0.409	38	20	19.166	6.531	0.933	0.400
STR23	322	6	3.455	0.003	0.600	0.533	67	7	6.845	0.159	0.795	0.206	39	11	10.746	4.072	0.869	0.343
STR24	333	5	3.874	0.000	0.442	0.258	79	3	3.000	0.000	0.535	0.143	35	7	6.874	3.932	0.745	0.378
STR25	267	3	2.996	0.001	0.551	0.809	82	6	5.073	0.076	0.489	0.699	34	9	8.735	3.813	0.743	0.398
STR26	339	7	6.226	0.000	0.699	0.227	91	13	10.077	0.207	0.842	0.173	39	16	15.293	5.105	0.917	0.065
STR27	338	7	6.096	1.008	0.580	0.377	90	13	11.307	2.279	0.881	0.112	39	11	10.672	1.568	0.854	0.270
STR28	304	6	5.873	0.001	0.716	0.526	88	8	6.661	0.022	0.669	0.231	38	15	14.709	7.223	0.909	0.238
STR29	300	16	8.589	0.699	0.610	0.365	80	18	14.442	1.986	0.880	0.228	34	24	23.185	10.752	0.956	0.250
STR30	336	4	3.999	0.000	0.551	0.280	89	8	6.200	0.996	0.753	0.205	39	10	9.547	4.345	0.853	0.057
STR31	339	9	6.320	0.075	0.666	0.295	82	12	9.244	1.479	0.767	0.105	39	16	14.753	6.076	0.892	0.097
STR32	334	8	5.187	0.000	0.715	0.363	68	7	6.992	0.907	0.798	0.201	39	14	13.494	7.254	0.901	0.049
STR33	335	9	7.094	0.033	0.677	0.237	91	14	12.686	0.385	0.900	0.177	39	15	14.374	4.045	0.925	0.185
STR34	183	5	5.044	0.032	0.635	0.309	39	7	7.684	0.666	0.819	0.334	27	7	6.969	1.040	0.770	0.216
STR35	179	2	1.336	0.000	0.017	-0.008	39	4	4.724	0.002	0.696	0.366	28	7	6.994	2.272	0.784	0.212
STR36	287	5	3.960	0.017	0.582	0.310	81	10	7.484	1.979	0.663	0.175	38	11	10.894	4.799	0.844	0.242
STR37	253	7	6.420	0.050	0.587	0.535	88	7	6.083	0.282	0.784	0.271	38	8	7.779	2.641	0.790	0.527
STR38	332	4	2.187	0.000	0.483	0.363	88	7	5.991	0.033	0.750	0.192	38	9	8.929	2.975	0.809	0.078
STR39	284	5	3.578	0.405	0.495	0.394	60	4	3.769	0.769	0.661	0.695	37	11	10.024	6.851	0.771	0.147
STR40	151	10	6.134	0.179	0.682	0.230	33	8	8.426	0.763	0.839	0.193	28	10	9.873	1.917	0.781	0.394
STR41	186	4	2.595	0.000	0.084	0.291	39	5	4.982	0.002	0.740	0.262	29	7	6.993	1.791	0.762	0.263
STR42	175	2	1.749	0.000	0.056	0.794	36	4	3.671	0.911	0.160	-0.059	27	5	5.000	2.211	0.433	0.215
STR43	183	7	5.443	0.000	0.690	0.190	39	9	9.200	1.345	0.753	0.068	28	15	15.752	7.946	0.878	0.254
STR44	187	7	6.022	0.000	0.779	0.222	38	8	7.704	0.096	0.812	0.343	29	11	10.817	2.028	0.869	0.233
STR45	317	8	5.954	0.000	0.626	0.313	89	11	9.276	0.047	0.836	0.108	39	11	10.857	1.481	0.850	0.144
**Mean**	296	6.4	4.798	0.149	0.581	0.368	77.6	8.4	7.266	0.666	0.723	0.209	36.5	11	10.68	4.11	0.825	0.219
**SE**	8.280	0.372	0.242	0.039	0.027	0.025	2.775	0.521	0.396	0.116	0.022	0.024	0.607	0.631	0.593	0.429	0.014	0.022

(*n*) Sample size per locus; (*Na*) Number of observed Alleles; (*Ar*) Allelic Richness; (*Par*) Private Allelic Richness; (*He*) Expected Heterozygosity; (F) Fixation Index
