# Supplementary material for: Levels and Patterns of Genetic Diversity and Population Structure in Domestic Rabbits
Source: PLoS One. 2015 Dec 21;10(12):e0144687. doi: 10.1371/journal.pone.0144687 (PMC4686922; doi:10.1371/journal.pone.0144687)
Supplement: S9 Table — (PDF) [file pone.0144687.s017.pdf]

S9 Table

	AN	BH	CH	CS	EN	SL	FB	FG	HG	FL	HI	ND	NZ	RX	TH	VW
**AN**	0.00000															
**BH**	0.30475	0.00000														
**CH**	0.24827	0.28054	0.00000													
**CS**	0.18569	0.25252	0.19995	0.00000												
**EN**	0.2745	0.36326	0.27613	0.2324	0.00000											
**SL**	0.21664	0.34509	0.2649	0.18406	0.25712	0.00000										
**FB**	0.23749	0.25341	0.19773	0.16095	0.24022	0.23948	0.00000									
**FG**	0.25617	0.3139	0.2458	0.18698	0.28153	0.25681	0.19251	0.00000								
**HG**	0.24457	0.32262	0.21371	0.15478	0.27042	0.2311	0.1823	0.09208	0.00000							
**FL**	0.22441	0.29324	0.1941	0.13393	0.22916	0.19119	0.13844	0.16054	0.13119	0.00000						
**HI**	0.25355	0.36376	0.27447	0.22503	0.29325	0.29371	0.24575	0.27709	0.27881	0.28376	0.00000					
**ND**	0.15059	0.26348	0.16944	0.11383	0.2588	0.23964	0.17049	0.15176	0.14633	0.13934	0.20389	0.00000				
**NZ**	0.23398	0.292	0.25625	0.19415	0.29038	0.27163	0.21615	0.24322	0.20553	0.22313	0.30771	0.16688	0.00000			
**RX**	0.16672	0.25182	0.15703	0.15769	0.22171	0.18362	0.13248	0.19516	0.16342	0.14792	0.19981	0.11141	0.14621	0.00000		
**TH**	0.22675	0.34687	0.26796	0.21656	0.24906	0.22127	0.2234	0.24061	0.20888	0.18533	0.30436	0.2205	0.27285	0.20452	0.00000	
**VW**	0.21028	0.28245	0.19559	0.15135	0.26028	0.21976	0.20867	0.17351	0.18227	0.17396	0.25094	0.14586	0.22915	0.17328	0.21127	0.00000

$P < 0.001$; Breed names correspondence in S1 Table
