# Supplementary material for: Levels and Patterns of Genetic Diversity and Population Structure in Domestic Rabbits
Source: PLoS One. 2015 Dec 21;10(12):e0144687. doi: 10.1371/journal.pone.0144687 (PMC4686922; doi:10.1371/journal.pone.0144687)
Supplement: S10 Table — Values for breed formation process were averaged across breeds and for each individual breed. Values were estimated using a resampling methodology (described in the Methods section). (PDF) [file pone.0144687.s018.pdf]

S10 Table

	Diversity Lost	Percentile (0.025)	Percentile (0.975)
**Colonization of France**	0.119735551	0.196316068	0.03938091
**Domestication**	0.207521514	0.266903081	0.139580829
**Breed Formation**	0.233260668	0.270653008	0.194174655
**French Angora**	0.218491619	0.266286536	0.165705799
**Belgian Hare**	0.367468536	0.414643896	0.322296434
**Chinchilla**	0.172281893	0.22016687	0.118318053
**Champagne Silver**	0.211144697	0.255568575	0.162595646
**English Spot**	0.267927139	0.313709956	0.219602009
**Fauve de Bourgogne**	0.226331714	0.290593977	0.163042836
**Flemish Giant**	0.264709746	0.312399981	0.217078819
**French Lop**	0.186886291	0.236565548	0.13650804
**Hungarian Giant**	0.25219862	0.323996951	0.180933547
**Himalayan**	0.266894467	0.315002899	0.214520755
**Netherland Dwarf**	0.171611151	0.223455786	0.11689325
**New Zealand**	0.347470212	0.385070444	0.306534431
**Rex**	0.130973428	0.178345167	0.081313445
**English Silver**	0.210269344	0.275633532	0.143667483
**Thuringer**	0.242597603	0.300709443	0.186639589
**Vienna White**	0.194914234	0.246022019	0.139077612
