# Supplementary material for: Levels and Patterns of Genetic Diversity and Population Structure in Domestic Rabbits
Source: PLoS One. 2015 Dec 21;10(12):e0144687. doi: 10.1371/journal.pone.0144687 (PMC4686922; doi:10.1371/journal.pone.0144687)
Supplement: S11 Table — (PDF) [file pone.0144687.s019.pdf]

S11 Table

K	Mean Similarity Coefficient
K=2	0.2583
K=3	0.1975
K=4	0.1543
K=5	0.2729
K=6	0.2439
K=7	0.2191
K=8	0.3841
K=9	0.3734
