# Supplementary material for: Levels and Patterns of Genetic Diversity and Population Structure in Domestic Rabbits
Source: PLoS One. 2015 Dec 21;10(12):e0144687. doi: 10.1371/journal.pone.0144687 (PMC4686922; doi:10.1371/journal.pone.0144687)
Supplement: S12 Table — (PDF) [file pone.0144687.s020.pdf]

S12 Table

	AN			BH			CH			CS			EN			FB			FG			FL		
**Markers**	**DF**	**ChiSq**	**P**	**DF**	**ChiSq**	**P**	**DF**	**ChiSq**	**P**	**DF**	**ChiSq**	**P**	**DF**	**ChiSq**	**P**	**DF**	**ChiSq**	**P**	**DF**	**ChiSq**	**P**	**DF**	**ChiSq**	**P**
STR01	3	5.373	0.146	1	3.857	0.050	3	7.408	0.060	10	27.592	0.002	6	7.122	0.310	6	8.213	0.223	3	0.734	0.865	6	5.899	0.435
STR02	15	21.109	0.133	3	17.851	4.72E-04	6	31.709	1.86E-05	6	19.917	0.003	10	12.600	0.247	6	8.071	0.233	15	23.086	0.082	15	47.212	3.41E-05
STR03	10	7.874	0.641	3	2.205	0.531	3	0.468	0.926	1	0.010	0.919	1	0.050	0.823	3	6.722	0.081	6	1.041	0.984	10	44.926	2.24E-06
STR04	6	2.943	0.816	3	1.250	0.741	3	18.509	3.45E-04	6	4.745	0.577	6	20.370	0.002	1	8.000	0.005	3	3.921	0.270	10	24.460	0.006
STR05	3	1.563	0.668	6	3.860	0.696	3	8.469	0.037	3	7.751	0.051	3	2.850	0.415	1	0.00	1.000	3	12.713	0.005	3	6.509	0.089
STR06	3	0.779	0.854	3	2.663	0.447	10	43.068	4.84E-06	3	7.478	0.058	6	0.801	0.992	1	0.036	0.850	6	13.184	0.040	3	28.189	3.32E-06
STR07	1	1.392	0.238	M	M	M	6	4.073	0.667	3	0.907	0.824	1	2.926	0.087	1	0.036	0.850	M	M	M	3	0.663	0.882
STR08	10	8.288	0.601	3	2.432	0.488	1	0.247	0.619	3	1.195	0.754	6	1.924	0.927	6	8.595	0.198	6	33.620	7.97E-06	3	0.405	0.939
STR09	21	55.501	5.97E-05	1	0.114	0.736	15	40.644	3.62E-04	10	8.847	0.547	6	1.563	0.955	6	6.580	0.361	21	16.113	0.763	15	14.705	0.473
STR10	1	2.778	0.096	1	0.013	0.909	1	0.013	0.909	3	0.107	0.991	3	0.465	0.927	1	8.000	0.005	3	0.549	0.908	M	M	M
STR11	1	2.667	0.102	6	3.360	0.762	1	1.976	0.160	3	6.349	0.096	1	0.00	1.000	3	0.889	0.828	3	0.889	0.828	1	0.309	0.579
STR12	6	4.656	0.589	3	4.492	0.213	6	9.993	0.125	10	14.708	0.143	6	3.122	0.793	1	0.036	0.850	6	3.180	0.786	6	6.053	0.417
STR13	1	0.060	0.807	1	3.070	0.080	3	13.910	0.003	3	5.914	0.116	6	25.889	0.000	3	8.222	0.042	1	0.549	0.459	M	M	M
STR14	3	0.490	0.921	3	0.710	0.871	6	10.686	0.099	6	7.844	0.250	6	18.342	0.005	3	4.475	0.215	3	8.750	0.033	3	0.757	0.860
STR15	6	3.044	0.803	1	0.247	0.619	3	1.315	0.726	3	17.275	0.001	6	1.541	0.957	3	0.426	0.935	1	0.583	0.445	3	4.765	0.190
STR16	3	4.152	0.245	1	0.233	0.630	6	1.830	0.935	3	10.757	0.013	3	5.929	0.115	3	1.680	0.641	6	11.756	0.068	6	16.082	0.013
STR17	6	3.140	0.791	6	2.051	0.915	3	4.357	0.225	6	12.391	0.054	6	0.309	0.999	6	4.959	0.549	3	1.280	0.734	6	3.742	0.712
STR18	6	5.112	0.530	3	1.833	0.608	3	0.815	0.846	6	1.205	0.977	3	3.090	0.378	1	0.036	0.850	6	1.544	0.957	3	1.004	0.800
STR19	1	5.000	0.025	3	8.000	0.046	1	0.202	0.653	10	64.622	4.79E-10	1	0.309	0.579	6	19.455	0.003	3	11.452	0.010	1	8.746	0.003
STR20	6	5.195	0.519	3	0.466	0.926	1	2.813	0.094	3	0.913	0.822	3	9.751	0.021	3	3.955	0.266	3	1.367	0.713	3	3.204	0.361
STR21	3	5.600	0.133	1	0.247	0.619	3	4.306	0.230	6	5.280	0.508	3	1.019	0.797	3	2.000	0.572	3	2.541	0.468	1	0.017	0.898
STR22	M	M	M	1	20.000	0.000	M	M	M	M	M	M	1	25.000	5.73E-07	1	8.000	0.005	M	M	M	M	M	M
STR23	1	0.010	0.922	3	5.548	0.136	3	3.756	0.289	3	6.369	0.095	1	0.104	0.747	1	8.000	0.005	6	2.690	0.847	6	5.556	0.475
STR24	3	7.482	0.058	1	0.012	0.911	3	11.555	0.009	1	0.644	0.422	1	0.887	0.346	1	0.036	0.850	3	0.327	0.955	1	1.989	0.158
STR25	3	18.296	3.82E-04	3	21.250	9.34E-05	3	14.612	0.002	1	18.113	2.08E-05	1	21.000	4.59E-06	3	9.742	0.021	3	11.136	0.011	3	31.529	6.58E-07
STR26	3	0.854	0.837	15	10.861	0.762	1	0.002	0.964	3	1.291	0.731	3	8.877	0.031	6	13.338	0.038	3	0.549	0.908	3	2.372	0.499
STR27	1	0.907	0.341	6	21.065	0.002	6	10.863	0.093	3	18.596	3.31E-04	6	8.292	0.217	1	0.163	0.686	3	3.374	0.337	3	3.243	0.356
STR28	1	8.543	0.003	M	M	M	10	57.423	1.11E-08	10	32.450	3.37E-04	3	8.884	0.031	1	1.215	0.270	6	16.047	0.014	10	88.667	9.84E-15
STR29	10	30.827	0.001	10	43.877	3.46E-06	21	41.691	0.005	3	38.218	2.54E-08	3	1.941	0.585	3	2.880	0.410	3	0.090	0.993	15	10.549	0.784
STR30	3	1.187	0.756	3	0.233	0.972	3	1.150	0.765	6	7.214	0.301	6	10.223	0.116	1	0.889	0.346	3	2.332	0.506	3	7.711	0.052
STR31	10	14.287	0.160	M	M	M	3	3.194	0.363	6	8.820	0.184	6	5.678	0.460	3	2.320	0.509	1	0.108	0.743	6	3.086	0.798
STR32	6	2.682	0.848	6	4.453	0.616	1	0.299	0.585	10	12.217	0.271	6	3.825	0.700	3	0.163	0.983	1	0.640	0.424	10	12.461	0.255
STR33	6	4.660	0.588	6	12.416	0.053	6	5.519	0.479	10	19.686	0.032	6	3.629	0.727	3	8.889	0.031	10	9.391	0.495	10	10.085	0.433
STR34	6	3.430	0.753	3	0.625	0.891	10	10.317	0.413	6	6.157	0.406	3	5.260	0.154	3	6.000	0.112	3	12.027	0.007	3	1.222	0.748
STR35	M	M	M	M	M	M	M	M	M	M	M	M	M	M	M	M	M	M	1	0.311	0.577	M	M	M
STR36	1	0.362	0.548	3	6.700	0.082	1	0.870	0.351	3	2.654	0.448	3	1.340	0.720	3	7.280	0.063	1	0.286	0.593	1	0.247	0.619
STR37	1	0.045	0.831	3	2.766	0.429	3	18.153	4.09E-04	6	35.779	3.04E-06	M	M	M	3	7.143	0.067	6	1.041	0.984	6	7.848	0.249
STR38	1	0.465	0.495	1	1.562	0.211	1	0.014	0.906	1	0.00	1.000	1	3.537	0.060	1	0.130	0.719	1	0.185	0.667	3	0.860	0.835
STR39	3	4.150	0.246	3	6.980	0.073	6	10.790	0.095	3	3.280	0.350	3	8.803	0.032	M	M	M	3	0.401	0.940	1	0.124	0.724
STR40	3	1.522	0.677	10	16.320	0.091	3	10.440	0.015	3	3.317	0.345	1	8.113	0.004	3	2.000	0.572	10	21.353	0.019	6	2.691	0.846
STR41	1	0.013	0.909	M	M	M	3	2.036	0.565	M	M	M	M	M	M	1	0.750	0.386	M	M	M	1	0.023	0.880
STR42	M	M	M	M	M	M	1	5.049	0.025	M	M	M	M	M	M	M	M	M	1	10.000	0.002	M	M	M
STR43	3	1.768	0.622	1	1.111	0.292	3	1.124	0.771	10	21.185	0.020	3	4.462	0.216	3	0.750	0.861	3	1.053	0.788	6	6.576	0.362
STR44	10	14.541	0.150	10	12.585	0.248	3	1.625	0.654	6	5.168	0.522	6	3.377	0.760	1	0.750	0.386	6	3.545	0.738	6	8.000	0.238
STR45	6	2.570	0.861	3	3.273	0.351	6	15.183	0.019	10	10.935	0.363	3	4.171	0.244	3	1.092	0.779	6	4.084	0.665	M	M	M

M –Represents monomorphic loci

	HG			HI			ND			NZ			RX			SL			TH			VW		
**Markers**	**DF**	**ChiSq**	**P**	**DF**	**ChiSq**	**P**	**DF**	**ChiSq**	**P**	**DF**	**ChiSq**	**P**	**DF**	**ChiSq**	**P**	**DF**	**ChiSq**	**P**	**DF**	**ChiSq**	**P**	**DF**	**ChiSq**	**P**
STR01	10	4.177	0.939	15	15.706	0.402	3	0.342	0.952	10	30.485	0.001	3	17.162	0.001	10	9.163	0.517	3	4.105	0.250	10	10.414	0.405
STR02	3	8.595	0.035	10	14.208	0.164	10	20.052	0.029	3	26.155	8.85E-06	6	14.038	0.029	10	22.748	0.012	3	3.129	0.372	6	8.519	0.202
STR03	3	1.653	0.647	M	M	M	3	8.610	0.035	10	26.265	0.003	3	4.867	0.182	1	8.146	0.004	1	0.023	0.880	1	0.019	0.890
STR04	3	0.747	0.862	10	34.099	1.78E-04	6	3.441	0.752	15	26.593	0.032	3	10.489	0.015	15	24.755	0.053	1	0.138	0.710	6	22.742	0.001
STR05	3	3.339	0.342	3	4.340	0.227	3	3.445	0.328	3	1.907	0.592	1	1.967	0.161	3	0.564	0.905	3	11.111	0.011	3	8.303	0.040
STR06	3	12.000	0.007	3	11.219	0.011	3	38.000	2.83E-08	10	41.501	9.20E-06	6	35.307	3.76E-06	6	27.154	1.35E-04	6	16.033	0.014	3	9.111	0.028
STR07	1	0.050	0.824	6	4.044	0.671	3	13.596	0.004	3	0.324	0.955	6	5.996	0.424	10	5.532	0.853	10	10.353	0.410	3	1.316	0.725
STR08	3	0.889	0.828	6	6.308	0.390	1	0.048	0.827	6	8.106	0.230	3	2.474	0.480	10	25.372	0.005	1	0.245	0.621	3	2.162	0.539
STR09	21	19.501	0.553	10	13.373	0.204	21	24.013	0.292	36	27.907	0.831	10	34.098	0.000	21	31.103	0.072	10	7.031	0.723	15	17.286	0.302
STR10	M	M	M	3	0.587	0.899	3	1.153	0.764	1	0.011	0.915	1	0.007	0.935	1	0.663	0.416	1	6.703	0.010	1	0.019	0.890
STR11	M	M	M	1	1.361	0.243	M	M	M	1	0.324	0.569	3	2.848	0.416	3	4.505	0.212	1	0.077	0.782	1	0.219	0.640
STR12	6	4.089	0.665	6	2.511	0.867	6	10.242	0.115	10	11.178	0.344	6	10.078	0.121	6	21.988	0.001	3	2.109	0.550	6	9.045	0.171
STR13	3	0.816	0.846	3	0.102	0.992	3	13.865	0.003	3	2.389	0.496	6	5.098	0.531	3	2.424	0.489	3	0.520	0.914	1	0.035	0.852
STR14	6	2.691	0.846	10	26.311	0.003	M	M	M	3	11.757	0.008	3	6.037	0.110	3	9.866	0.020	3	0.960	0.811	3	4.653	0.199
STR15	3	3.061	0.382	6	6.874	0.333	1	0.518	0.472	3	3.267	0.352	3	0.454	0.929	3	2.722	0.437	6	1.170	0.978	3	6.238	0.101
STR16	6	16.000	0.014	6	4.029	0.673	3	2.429	0.488	6	38.300	9.81E-07	3	28.493	2.86E-06	1	25.000	5.73E-07	M	M	M	3	4.927	0.177
STR17	3	4.840	0.184	3	1.999	0.573	10	11.851	0.295	10	17.674	0.061	3	1.422	0.700	10	20.197	0.027	1	0.066	0.797	M	M	M
STR18	1	0.130	0.719	3	2.147	0.543	6	9.686	0.139	3	3.694	0.296	1	0.637	0.425	3	4.998	0.172	1	0.221	0.638	1	1.260	0.262
STR19	6	4.375	0.626	10	47.354	8.14E-07	3	10.259	0.016	6	6.214	0.400	1	2.298	0.130	3	26.729	6.71E-06	3	12.715	0.005	6	11.600	0.072
STR20	3	0.163	0.983	6	3.332	0.766	1	1.252	0.263	6	6.376	0.382	3	4.199	0.241	3	13.052	0.005	3	2.528	0.470	10	7.795	0.649
STR21	1	0.194	0.659	3	10.494	0.015	3	7.194	0.066	3	20.135	1.59E-04	1	0.009	0.926	3	3.680	0.298	3	3.644	0.303	1	0.585	0.444
STR22	M	M	M	1	0.189	0.664	1	7.887	0.005	M	M	M	6	59.823	4.89E-11	1	13.405	2.51E-04	M	M	M	M	M	M
STR23	3	7.006	0.072	10	15.268	0.123	1	0.011	0.915	1	2.358	0.125	6	39.532	5.63E-07	3	13.512	0.004	3	7.718	0.052	1	5.915	0.015
STR24	3	0.426	0.935	3	0.965	0.810	3	2.066	0.559	1	1.775	0.183	3	0.923	0.820	3	13.007	0.005	1	12.000	0.001	1	2.431	0.119
STR25	3	6.240	0.100	3	13.361	0.004	1	0.110	0.740	1	11.000	0.001	1	30.126	4.05E-08	3	24.542	1.93E-05	3	20.000	1.70E-04	1	6.043	0.014
STR26	1	0.036	0.850	10	14.799	0.140	3	0.781	0.854	10	6.866	0.738	3	7.665	0.053	6	5.698	0.458	6	8.616	0.196	3	5.661	0.129
STR27	1	0.426	0.514	10	11.905	0.291	6	17.505	0.008	1	0.897	0.344	1	2.036	0.154	3	20.655	1.24E-04	1	1.170	0.279	6	15.831	0.015
STR28	3	7.778	0.051	10	27.327	0.002	10	45.997	1.44E-06	3	36.000	7.49E-08	10	64.474	5.11E-10	6	48.022	1.17E-08	10	34.757	1.37E-04	15	45.222	7.06E-05
STR29	3	0.918	0.821	1	0.548	0.459	3	10.961	0.012	21	52.500	1.62E-04	15	71.128	2.81E-09	6	9.170	0.164	6	0.430	0.999	3	12.600	0.006
STR30	6	4.667	0.587	6	11.279	0.080	6	2.339	0.886	6	3.834	0.699	1	11.788	0.001	6	15.355	0.018	1	0.008	0.928	1	2.431	0.119
STR31	3	2.889	0.409	6	12.531	0.051	10	5.113	0.884	10	11.239	0.339	3	3.288	0.349	10	5.260	0.873	6	1.868	0.931	6	2.580	0.859
STR32	6	3.701	0.717	6	4.259	0.642	3	22.014	6.48E-05	6	16.200	0.013	15	99.951	1.33E-14	3	31.119	8.02E-07	3	0.537	0.911	6	4.467	0.614
STR33	6	18.469	0.005	10	11.566	0.315	15	18.152	0.255	10	4.408	0.927	3	3.558	0.313	6	3.656	0.723	6	4.230	0.646	6	1.041	0.984
STR34	6	5.533	0.477	3	19.210	2.47E-04	6	2.639	0.853	6	7.375	0.288	1	0.014	0.906	3	0.747	0.862	6	5.625	0.466	3	4.548	0.208
STR35	M	M	M	M	M	M	M	M	M	M	M	M	M	M	M	M	M	M	M	M	M	M	M	M
STR36	1	0.889	0.346	6	4.114	0.661	3	0.626	0.891	6	3.468	0.748	3	2.395	0.495	1	0.00	1.000	1	0.050	0.824	3	4.083	0.253
STR37	10	8.500	0.580	6	5.590	0.471	1	0.025	0.875	6	0.107	1.000	1	0.045	0.831	1	4.041	0.044	1	0.194	0.659	3	11.224	0.011
STR38	1	2.782	0.095	1	0.465	0.495	1	2.457	0.117	3	8.903	0.031	1	1.072	0.301	1	3.074	0.080	1	0.025	0.875	1	0.389	0.533
STR39	3	1.120	0.772	1	0.011	0.917	3	22.348	5.52E-05	1	5.284	0.022	3	17.978	4.44E-04	1	0.010	0.919	1	10.000	0.002	6	6.842	0.336
STR40	6	5.000	0.544	6	5.756	0.451	10	15.833	0.105	1	0.031	0.860	6	0.389	0.999	1	0.889	0.346	3	3.333	0.343	1	0.257	0.613
STR41	M	M	M	M	M	M	M	M	M	3	1.907	0.592	M	M	M	M	M	M	M	M	M	M	M	M
STR42	M	M	M	M	M	M	1	12.000	0.001	M	M	M	M	M	M	M	M	M	1	1.852	0.174	M	M	M
STR43	3	0.313	0.958	10	24.440	0.007	10	18.833	0.042	10	5.857	0.827	6	7.650	0.265	6	2.556	0.862	1	1.500	0.221	3	1.233	0.745
STR44	6	5.556	0.475	6	14.877	0.021	3	1.466	0.690	10	13.719	0.186	10	13.784	0.183	10	13.469	0.199	10	12.587	0.248	3	1.519	0.678
STR45	M	M	M	6	17.632	0.007	15	19.898	0.176	6	5.909	0.433	6	14.355	0.026	6	5.480	0.484	1	0.025	0.875	1	0.083	0.773

M –Represents monomorphic loci
